# Porous metal-metalloporphyrin gel as catalytic binding pocket for highly efficient synergistic catalysis

**DOI:** 10.1038/s41467-019-09881-9

**Published:** 2019-04-23

**Authors:** Weijie Zhang, James J. Dynes, Yongfeng Hu, Pingping Jiang, Shengqian Ma

**Affiliations:** 1 The Key Laboratory of Food Colloids and Biotechnology, School of Chemical and Material Engineering, Jiangnan University, Wuxi, 214122 China; 2 Department of Chemistry, University of South Florida, 4202 East Fowler Avenue, Tampa, FL 33620 USA; 3 Canadian Light Source, 44 Innovation Boulevard, Saskatoon, Saskatchewan S7N 2V3 Canada

**Keywords:** Asymmetric catalysis, Heterogeneous catalysis, Chemical bonding

## Abstract

Synergistic catalysis occurring in an enzyme pocket shows enhanced performance through supramolecular recognition and flexibility. This study presents an aerogel capable of similar function by fabricating a gel catalyst with hierarchical porosity. Here, the as-prepared Co-MMPG, a Co(II) metal-metalloporphyrin gel, maintains enough conformational flexibility and features a binding pocket formed from the co-facial arrangement of the porphyrin rings, as elucidated through the combined studies of solid-state NMR and X-ray absorption near-edge structure (XANES). The cooperativity between two Co(II) sites within the defined nanospace pocket facilitates the binding of different substrates with a favourable geometry thereby rendering Co-MMPG with excellent performance in the context of synergistic catalysis, especially for the kinetic control stereoselective reactions. Our work thus contributes a different enzyme-mimic design strategy to develop a highly efficient heterogeneous catalyst with high chemo/stereo selectivity.

## Abstract (web summary)

Synergistic catalysis occurring in an enzyme pocket shows enhanced performance through supramolecular recognition and flexibility. Here the authors design an enzyme-mimic strategy to develop a Co(II) metal-metalloporphyrin gel with excellent synergistically catalytic performance and chemo/stereo selectivity.

## Introduction

Synergistic catalysis, as widely adopted by enzymes, is ubiquitous in biological systems^1,2^. Enzymes achieve the synergy via induced fit, for which the pocket featuring multiple binding/recognition sites (preconcentration effects) and conformational flexibility are essential (orientation effects)^3–5^. Extensive yet continuous efforts have been dedicated to mimic enzymes for synergistic catalysis, and significant progress has been accomplished in creating active centers with binding/coordination environment similar to enzymes^6–8^. Nonetheless, it remains a challenge, particularly in the solid state, to combine both binding pocket and flexibility into one system to function like enzymes that feature high specificity, selectivity, and efficiency^9–11^.

Metal–organic gels (MOGs)^12–17^, a class of soft-hybrid polymer materials are functional porous aerogels that have characteristics of low density, versatile porosity, and high-internal surface area. These polymer materials have shown potential for applications in catalysis^18–21^, adsorption/separation^22,23^, and others^24,25^. Given their optimal flexibility, MOGs could offer some opportunities to mimic enzymes for synergistic catalysis superior to those of their homogeneous counterparts. To achieve synergistic activity in MOGs, we propose herein the construction of hierarchically porous MOGs that can be combined to show similar coordination found in metal–organic frameworks (MOFs)^26,27^, capable of mimicking the binding pocket/active center of enzyme yet show greater conformational flexibility similar to organic gels^28,29^. Therefore, MOGs are rigid but not too rigid, as well as flexible but not too flexible. In addition, a high density of binding sites combined with pre-orientations can engender a catalytic binding pocket that works concertedly to intensify the formation of relevant intermediate and, in turn, enhance catalytic performance including both conversion and chemo/stereo selectivity^11,30^. Considering its ubiquitousness in nature as a co-factor/active center of enzymes, we selected metalloporphyrin as the building block for proof of principle studies and the afforded metal–metalloporphyrin gel (MMPG) exhibits interesting synergistic catalysis as illustrated in the context of acyl-transfer^8^ and Diels–Alder reactions^31^.

**Fig. 1:**
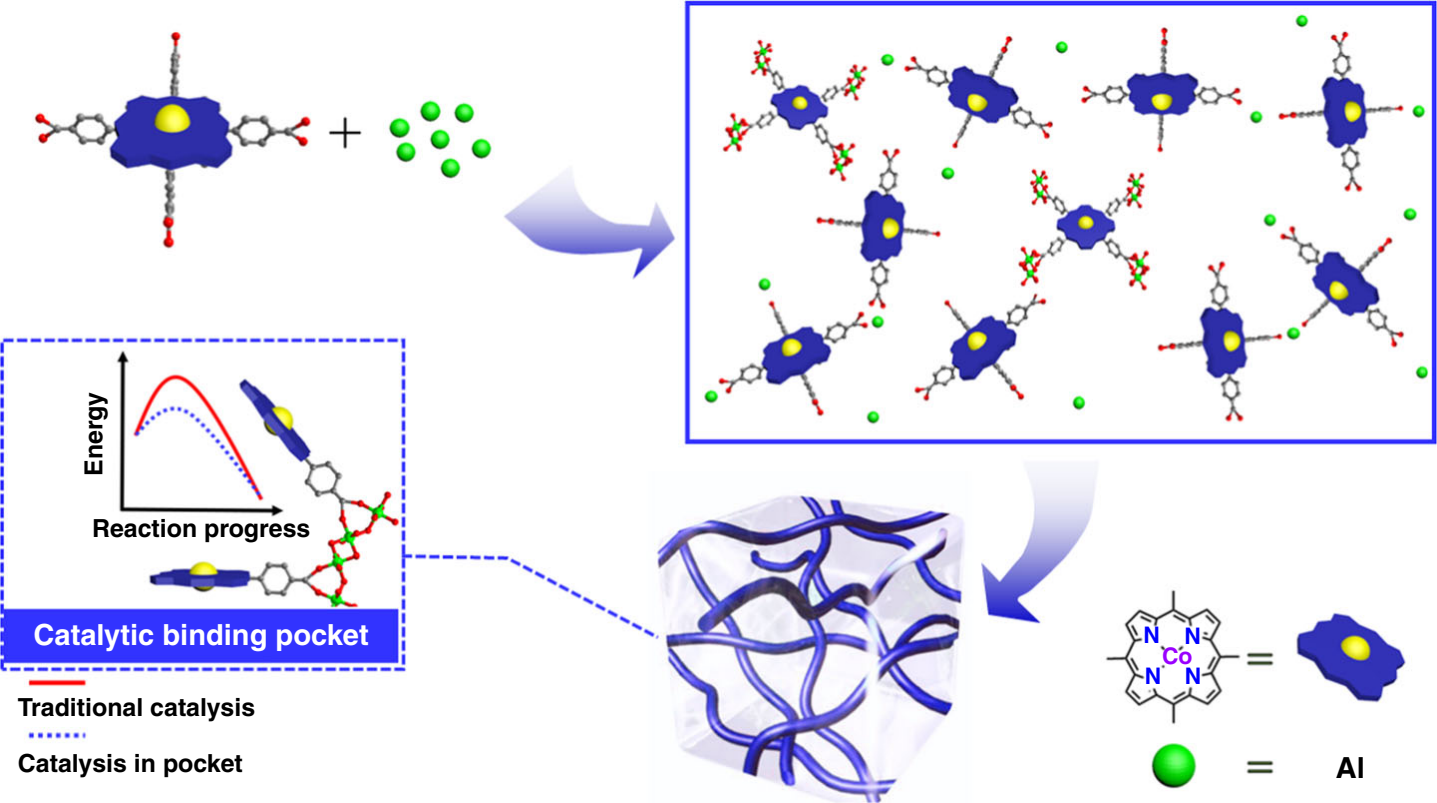
Schematic representation of the formation of Co-MMPG and its activation energy diagram with traditional catalysis and synergistic catalysis. MOGs engender a catalytic binding pocket that decreases the activation energy, and, in turn, enhance catalytic performance including both conversion and chemo/stereo selectivity

From a structural point of view, the key component in porphyrin assemblies for synergistic catalysis is the bonding connectivity between porphyrins. Because porphyrin materials in gel form can be designed with multiple and precisely spaced Lewis acid sites at a very high local concentration and exhibit conformational flexibility^32^, they are an ideal platform to engineer as recyclable solid catalysts for synergistic catalysis. In this contribution, we report a hierarchically porous metal–metalloporphyrin aerogel matrix by utilizing Co(II) Tetrakis(4-carboxyphenyl)porphyrin (Co-TCPP) as the building unit, named Co-MMPG (Fig. 1 and Supplementary Fig. 1). Co-MMPG demonstrates superior catalysis performance in terms of both activity and selectivity in comparison with the homogeneous metalloporphyrin, the microporous metalloporphyrin-containing polymer and the metalloporphyrin-based MOF counterpart, which can be primarily attributable to the synergistic effect of two Co(II) binding sites within the porphyrin binding pocket as a result of the inherent correlation between MOFs and MOGs with comparable bonding connectivity and conformational flexibility. In addition, the hierarchical porous structure of Co-MMPG with interconnected micropores and mesopores can facilitate the substrate transport thereby further promoting the catalytic processes.

## Results

### Structural characterization

**Fig. 2:**
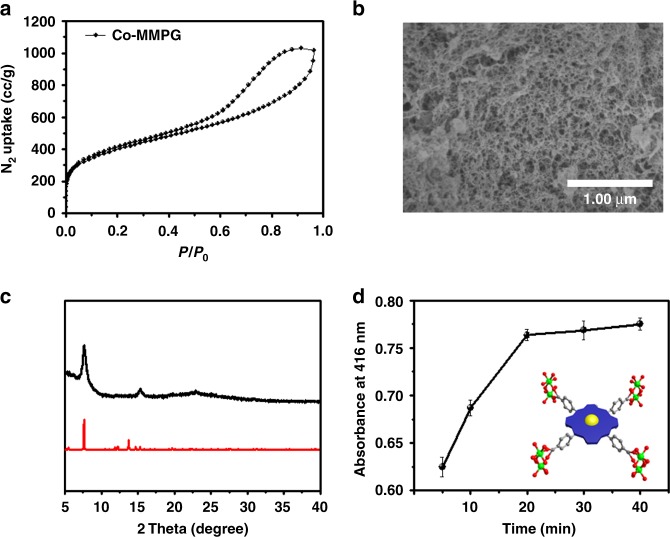
Structural characterization. **a** The N_2_ sorption isotherms of Co-MMPG at 77 K; **b** SEM of Co-MMPG after supercritical CO_2_ drying; **c** PXRD patterns: Co-MMPG after activation (up) and calculated from the corresponding MOF stucture; **d** monitoring the formation of porphyrin–Al cluster in the ethanol solutions of Co-TCPP and Al(NO_3_)_3_ by UV–vis spectra (the optical density at 415 nm slowly goes up within the increase of time). The S.E.M. bar represents the standard deviation from the repeated experiment after three times. Source data are provided as a Source Data file

The permanent porosity of Co-MMPG was confirmed by N_2_ sorption isotherms collected at 77 K (Fig. 2a), which reveals that Co-MMPG exhibits a Brunauer–Emmett–Teller (BET) surface are of 1343 m^2^ g^−1^ (*P*/*P*_0_ = 0.0001–0.1), corresponding to a Langmuir surface area of ~1678 m^2^ g^−1^. It is worth noting that Co-MMPG exhibits a sorption behavior of type I with a sharp uptake of N_2_ at low-relative pressure (*P/P*_0_ < 0.1) plus type IV with a hysteresis loop at higher relative pressure (0.4 < *P/P*_0_ < 0.8). Calculated from the nonlocal density functional theory method, the pore sizes of Co-MMPG are distributed predominantly at 1.4, and 2.7–17 nm, indicating that the hierarchical porous Co-MMPG mainly consists of micropores and mesopores (Supplementary Fig. 2).

Field-emission scanning electron microscopy (SEM) images show that Co-MMPG is composed of cross-linked nanofibers with sizes ranging from 200 to 300 nm in diameter (Fig. 2b). The energy-dispersive spectroscopy analysis indicates that the Co(II) is uniformly distributed in Co-MMPG (Supplementary Fig. 3). Also, particles in aggregates could not be detected in transmission electron microscopy (TEM) (Supplementary Fig. 4). Inductively coupled plasma mass spectrometry together with elemental analysis indicated that the Co content is 1.0 wt%. Co-MMPG is stable up to 380 ^o^C and its decomposition starts at or after 380 ^o^C (Supplementary Fig. 5). For Co-TCPP, the absorption bands centered at 1704 cm^−1^ were observed, which can be attributed to the noncoordinated COOH groups. For Co-MMPG, the strength of COOH groups greatly decreases, which indicates that COOH group coordinates with Al after the gel formation (Supplementary Fig. 6).

We systematically explored the gelation behavior of Co-MMPG, which is reminiscent of the occasional observations of gel formation in traditional porphyrin-based and other related gel syntheses^33–35^. The formation of Co-MMPG can be considered to evolve from two main stages. In the early stage of the formation, a stronger ligand–metal coordination relative to the solvent–metal interactions drives the ligands and metal ions to assemble into porphyrin–Al clusters; in the subsequent stage, the porphyrin–Al clusters aggregate, which is followed by the lateral arrangement of these individual aggregates into microscale fibers. Such an evolving process can be evidenced by UV–vis and powder X-ray diffraction (PXRD) experiments (Fig. 2c, d). As shown in Fig. 2c, PXRD studies showed that Co-MMPG retained its structural integrity after activation; the broader peaks toward lower 2 theta values are due to the aggregates with conformational flexibility caused by twisting Al–O–Al cluster. In addition, a high value of ~6 was observed for the PXRD intensity ratio between the (2 0 1) to the (1 1 0) plane of the sample, which indicates that each individual porphyrin layer utilizes the strong π–π stacking interaction to interact with other porphyrin layers to extend along the *Z*-direction instead of *X-* and *Y-*directions (Supplementary Fig. 7) thereby allowing the continuous growth to form fibers^36^. We speculate that the twisting Al–O–Al cluster leads to the formation of an enzyme-like binding pocket via a spontaneously upward or downward incline of porphyrin plane, which is anticipated to significantly promote the rate of synergistic catalysis.

To gain a better insight into the metal coordination gelation process, the UV–vis absorption spectra of the Co-TCPP, Co-TCPP with Al(NO_3_)_3_ and Co-TPP with Al(NO_3_)_3_ were measured in ethanol at temperatures ranging from 20 to 80 ^o^C (Supplementary Figs. 8 and 9). The slight difference in the absorption maxima for monomer forms of Co-TCPP vs. Co-TCPP with Al(NO_3_)_3_ suggests that there is a slight different electronic interaction between the H bond system and metal coordination system of Co-porphyrin arrays (Supplementary Fig. 8). Upon increasing the temperature from 20 to 80 °C, the emission band diminished in intensity and a slight blue shift in the emission maxima was observed for only Co-TCPP in the process (Supplementary Fig. 9). The change in UV–vis absorption spectra for only Co-TCPP without Al(NO_3_)_3_ can be ascribed to the two-dimensional hydrogen-bonded network of Co-TCPP^26,34^. However, similar experiments employing ethanol solutions containing Co-TCPP and Al(NO_3_)_3_, revealed that the intensity at 415 nm slowly went up within increasing heating time up to 40 min, which indicated that COOH groups coordinate with Al after the gel formation (Fig. 2d, Supplementary Figs. 10, 11). This process is ascribed to the formation of ligand–Al cluster based aggregate. It is noteworthy that hydrogen bonds with dissociation energies cover the range between 12 and 120 kJ mol^−1^, obviously lower than the Al–O bond dissociation energy (∆*H*_298_ = 501.9 ± 10.6 kJ mol^−1^)^37,38^. In this regard, it is favorable for the metal coordination assisted aggregates to form at a higher temperature. For comparison, Supplementary Fig. 12 shows that there is no change in either the Soret or Q bands for Co-TPP with Al(NO_3_)_3_, indicating the role of the carboxylate group in the aggregation process. Interestingly, fresh Co-MMPG sample gives different PXRD patterns from the calculated ones of the single-crystal Co-porphyrin-based Al-MOF (denoted as Co-PMOF)^39^, even when the concentration reaches 1 M (Supplementary Fig. 13). This implies that there is no crystalline components or long-range order during the gelation process, in line with the TEM results.

The Co-MMPG skeleton is further verified by the solid-state ^27^Al nuclear magnetic resonance (NMR) spectra showing two peaks (Supplementary Fig. 14), which can be attributed to other coexisting Al(III)–TCPP motifs with short range order and mixed coordination environments^12,40^. In addition, the peak located around 70 ppm is attributed to existing tetrahedral Al(III)–porphyrin-solvent motifs. These Al species may bind partially with deprotonated Co-TCPP and some solvent molecules to form the one-dimensional inorganic chains of [Al(COO)_*n*_], which contribute to gelation by linking the Co-TCPP to afford the gel scaffold.

**Fig. 3:**
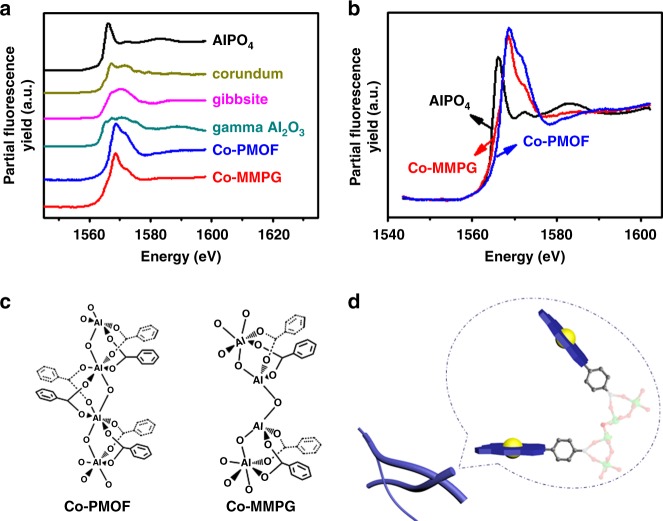
Coordination environment around Al(III). **a** Al K-edge XANES spectra of types of Al(III)-based samples, including AlPO_4_, corundum(*α*-Al_2_O_3_), gibbsite(Al(OH)_3_), gamma Al_2_O_3_(*γ*-Al_2_O_3_), Co-PMOF, and Co-MMPG; **b** Al K-edge XANES spectra of types of Al(III)-based samples including AlPO_4_, Co-PMOF, and Co-MMPG; **c** the local structure of Co-PMOF based on single X-ray data^39^ and Co-MMPG based on XANES spectra; **d** the proposed local structure of Co-MMPG. Source data are provided as a Source Data file

The coordination environment around the Al(III) in the Co-MMPG material was further investigated using Al K-edge X-ray absorption near-edge structure (XANES), which is sensitive to the electronic structure and the coordination environment around aluminum reported by our previous work^41^. As shown in Figs. 3a, b, Al containing compounds with tetrahedral coordinated Al, such as AlPO_4_, have a strong single-peak maximum at 1566.1 ± 0.7 eV and a weaker peak features at higher energy. Octahedrally coordinated Al, including in corundum (*α*-Al_2_O_3_) and gibbsite (Al(OH)_3_), have two main maxima at higher energies around 1567.8 ± 0.4 and 1571.5 ± 0.4 eV and the relative intensities of these two peaks can vary depending on the aluminum structure and the influence of the second neighbor^42^. Al containing compounds with both tetrahedrally and octahedrally coordinated Al (e.g., *γ*-Al_2_O_3_) have three main maxima at 1566.1 ± 0.3, 1567.3 ± 0.4, and 1570.5 ± 0.2 eV^42^. It is clear that the majority of the Al in Co-PMOF and Co-MMPG was octahedrally coordinated based on their two-peak profile around 1570 eV (Fig. 3a, b). However, the coordination environment around the Al was different in the two materials as evident from the peak positions and ratio of the octahedral peaks. Moreover, there appears to be an Al satellite peak centered at 1566.2 eV in Co-MMPG (Supplementary Fig. 15), suggesting that there was also some tetrahedrally coordinated Al in Co-MMPG (Fig. 3c, d)^43^. Such tetrahedrally coordinated Al species could render the flexibility and facilitate co-facial arrangement of the porphyrin rings in Co-MMPG.

### Investigation of catalysis performance

**Fig. 4:**
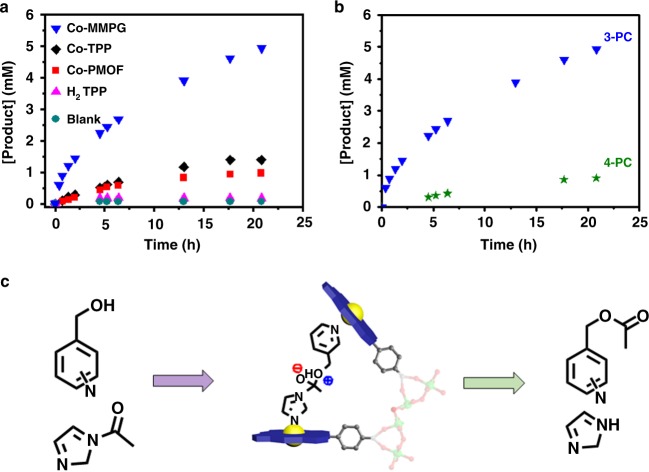
Catalytic property tests. **a** Kinetic profile of producing 3-acetoxymethylpyridines catalyzed by various catalysts; **b** kinetic profile of acryl-transfer reactions between N-acetylimidazole (NAI) and 3-pyridylcarbinol (3-PC) or 4-pyridylcarbinol (4-PC) catalyzed by Co-MMPG; **c** a possible local structure of the pocket, in which convergent binding sites are positioned in such a way that substrate molecules can be held in close proximity. Source data are provided as a Source Data file

In order to support co-facial arrangement of the porphyrin rings in Co-MMPG, the local pocket environment was investigated using acryl-transfer reactions. These reactions are very sensitive to the interactions between the structure of substrate isomers and the structure of catalyst’s active centers^8,44^. The acryl-transfer reaction between N-acetylimidezole (NAI) and 3-pyridylcarbinol (3-PC) producing 3-acetoxymethylpyridines (3-AMP) was carried out in acetonitrile at 50 ^o^C. Control experiments were conducted for homogeneous free-base H_2_TPP, cobalt(II)-metalated TPP (Co-TPP), Co-PMOF, and a blank under the same conditions. As shown in Fig. 4a, Co-MMPG demonstrated the most efficient catalytic activity for acryl-transfer of the compounds studied in terms of conversion (82% after 20 h). This compares favorably to the corresponding values for homogeneous H_2_TPP (3%), Co-TPP (23%), Co-PMOF (16%), and the blank (1%). Moreover, ~10-fold rate enhancement was observed for Co-MMPG compared to homogeneous Co-TPP. We reasoned that such dramatic enhancement of catalytic performance in Co-MMPG in comparison with Co-TPP and also Co-PMOF should stem from the favorable binding event in a bimolecular-like pocket formed from the co-facial arrangement of the porphyrin rings. No detectable leaching of active site and cobalt ions in the reaction solution were observed after removal of Co-MMPG by filtration. In addition, Co-MMPG can be reused for five cycles without significant drop in its catalytic activity (Supplementary Fig. 16). There was no obvious increase in conversion after a hot filtration of Co-MMPG (Supplementary Fig. 17). No morphological change was observed for Co-MMPG after reused (Supplementary Fig. 18) and the PXRD peak positions are almost the same for both the fresh and recycled Co-MMPG (Supplementary Fig. 19). These results indicate that acyl-transfer reactions catalyzed by Co-MMPG is heterogeneous catalysis.

By binding complementary pairs of NAI and 3-PC within the nanoscale pockets, which have a Co–Co separation, Co-MMPG could serve to align and orient the reactants in a fashion that is highly beneficial for the acryl-transfer reaction. Therefore, to gauge the significance of preferential substrate alignment, we then extended the study to 4-pyridylcarbinol (named as 4-PC). According to a previous report, 4-PC presents a different acyl orientation than 3-PC when bound to the Co site^7^. Fig. 4b shows that there is high sensitivity of the reaction rate to the substrate isomer structure, identical to that observed for supramolecular catalyst^45^. Such high-reaction rate sensitivity to the substrate isomer structure also excludes the parallel co-facial configuration as observed in porphyrin MOFs^7^. The abovementioned discussion allows us to propose a possible local structure of the pocket, in which convergent binding sites are positioned in such a way that substrate molecules can be held in close proximity (Fig. 4c).

The acyl-transfer reactions produce 3-AMP catalyzed by free base and other metallated MMPG catalysts (Supplementary Fig. 20) have also been investigated. Supplementary Fig. 21 shows the kinetic profile for the formation of 3-AMP catalyzed by Co-MMPG, Cu-MMPG, Mn-MMPG, and free-base MMPG, which suggests different activity of metal binding site in MMPG with Co(II) performing best^46^. Further examination of 3-AMP formation catalyzed by other Co(II)-based porphyrin MOF catalysts reveals the superior performance of Co-MMPG in comparison with PCN-222 and MOF-525 (Supplementary Figs. 22–24)^8^. Compared with the results of Co-TPP, the di-Co(II) Pacman porphyrins anchored by xanthene [Co_2_(DPX)] and dibenzofuran [Co_2_(DPD)]^47^ did not display enhanced catalytic performance (Supplementary Fig. 25). It has been previously reported that the highest initial rate for 3-PC with MOF-525 is about double that of the reported NU-902^8^. Therefore, it can be deduced that Co–Co distance of no less than 10 Å together with favorable orientation and proximal positioning of NAI and PC by metal sites favors the rate enhancement. The dimension of the binding pocket inside Co_2_(DPX) and Co_2_(DPD), especially the Co–Co distance (Supplementary Fig. 26) was anaylzed. The Co–Co distances of all porphyrin dimers in Co_2_(DPX) (3.7 Å) and Co_2_(DPD) (7.8 Å) are significantly shorter than the distances necessary for the intermediates. Furthermore, the Co content can be changed in a controlled manner via adjusting the ratio of Co-TCPP and TCPP during the gel formation process. Without the suitably oriented neighboring active sites, a slight reduction in Co content leads to a sharp decrease of catalytic efficiency (Supplementary Fig. 27). The rate constants have been extracted and compared with the porphyrin-based catalytic systems, such as homogeneous porphyrin monomer, dimer, trimer, microporous metalloporphyrin-containing polymer, and MOFs. An inferior activity has been observed in comparison with Co-MMPG, highlighting the importance of the local pocket structure with suitably oriented neighboring active sites (Supplementary Table 1).

## Discussion

**Fig. 5:**
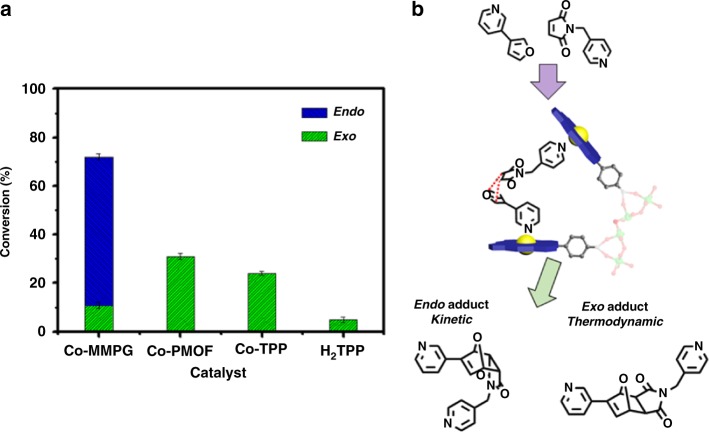
Catalytic performance for Diels–Alder reactions. Conversion and selectivity of exo and endo reactions in the presence of various catalysts (**a**) and Diels–Alder reaction between a diene and dienophile which are designed to react within Co-MMPG (**b**). The conversion and selectivity represent the standard deviation from the repeated experiment after three times. Source data are provided as a Source Data file

In order to further assess the merits of this catalyst, we examined the performance of Co-MMPG in Diels–Alder reaction as shown in Fig. 5. In the presence of Co-MMPG, a high catalytic activity was also observed with a yield of 72% and endo selectivity of 85% over 48 h. Interestingly, a dramatic decrease in the yield of the endo adduct and conversion was observed for Co-PMOF, Co-TPP, and H_2_TPP with a conversion of 31%, 24%, and 5%, respectively. An inferior activity was also observed for other gel catalysts of Cu-MMPG, Mn-MMPG, and free-base MMPG and the Co(II)-based porphyrin MOF catalysts of PCN-222 and MOF-525 in comparison with Co-MMPG (Supplementary Fig. 28). It has been reported that the endo adduct is usually the kinetic dominant product; however, the exo adduct tends to be the major product in reversible thermodynamically controlled reactions^31^. Therefore, we reasoned that the superior catalytic performances of Co-MMPG should result from the synergistic effect that the substrates bind in an orientation that closely approaches the endo transition state (Fig. 5b).

In summary, a porous metalloporphyrin-based metal–organic gel featuring a catalytic binding pocket to benefit synergistic catalysis has been achieved by combining cobalt porphyrin monomers with a twisting Al–O–Al cluster. As a result of the synergy between two Co(II) binding sites within defined nanospace pocket, Co-MMPG demonstrates excellent performance in the context of synergistic catalysis, especially for the kinetic control stereoselective reactions. Our work thereby not only provides a enzyme-mimic design approach to prepare highly active MOG-based catalysts but also lays a solid foundation for the development of metal–organic gel as a type of highly efficient heterogeneous catalyst that can merge synergistic catalysis and porous materials to carry out chemo/stereoselective chemistry.

## Methods

### Materials and measurements

All reagents were purchased in high purity grade from Fisher Scientific, Sigma-Aldrich, Alfa and used without further purification. Metal Tetrakis(4-carboxyphenyl)porphyrin (M-TCPP)^48^ were synthesized according to the procedures in references. 3-(3-Furyl)pyridine and 4-(Maleimidomethyl)pyridine were prepared by a published procedure^31^. 3-(3-Furyl)pyridine was eluted immediately afterward as a by-product and protected under N_2_. Solvents were purified according to standard methods and stored in the presence of molecular sieves. X-ray powder diffraction data were recorded on a Bruker D8 Advance X-ray diffractometer. Elemental analyses were performed on a Perkin-Elmer 2400 element analyzer. Gas sorption isotherms were measured on the Micrometrics ASAP 2020 Surface Area and Porosity Analyzer. UV–vis analysis were performed on a Varian CARY50 spectrophotometer. SEM measurements were performed on a CamScan CS44 scanning electron microscope. High-angle-annular-dark-field scanning STEM imaging, energy-dispersive X-ray spectroscopy mapping were carried out by Titan ChemiSTEM operated at 200 kV.

### X-ray absorption near-edge structure analysis

All the Al K-edge X-ray absorption near-edge structure (XANES) data were collected at the spherical grating monochromator beamline at the Canadian Light Source (Saskatoon, Canada)^49^. The energy scale of the Al K-edge spectra were calibrated using AlPO_4_ assuming a value of 1566.1 eV for the major peak^42^. The photon energy resolution was about 0.1 eV. The fine powders of the 1-G and 2-F and reference material (AlPO_4_) were spread on double-sided carbon tape. The partial fluorescence yield was measured using an Amptec silicon drift detector with an energy resolution of ∼150 eV. The Al spectra were normalized by simultaneously measuring the incident flux (*I*_0_) from an Au mesh (90% transmission) placed before the sample. All scans were acquired in a fast-scan mode (10 s per scan).

### Typical synthetic procedure of Co-MMPG

Co-TCPP (10 mg) and Al(NO_3_)_3_·9H_2_O (10 mg) were each dissolved in ethanol (0.5 mL), and the two solution were rapidly mixed in a short time. The resulting homogeneous red solution was allowed to stand at 353 K for gelation in a closed glass container. The Co-MMPG was formed after 60 min. After gelation, the wet gel was aged for another 3 days at same temperature. Subsequently, the obtained wet gel was subjected to a solvent exchange process using ethanol as solvent for 3 day using a Soxhlet extractor. The as-prepared gel was then placed into a high-pressure Soxhlet extractor (0.75 l). The solvent in the wet gel was slowly extracted with liquid CO_2_ (260–280 g) for 30 h, and the extraction temperature was strictly maintained at 308 K. Finally, the Co-MMPG product was obtained after carefully and slowly depressurizing the stainless-steel autoclave at room temperature for 3–4 h.

### Catalytic reactions for acyl-transfer reactions

Stock solutions in acetonitrile were made of N-acetylimidazole (6 mM); 3- and 4-PC (9 mM); and biphenyl (2 mM). Into a 15-dram screw-cap vial were combined acetonitrile (7 mL), biphenyl stock (1 mL), PC stock (1 mL), and Co-MMPG catalyst (9.3 mg, 10 mol%). The resulting mixture was stirred for 5 min before the NAI stock (1 mL) was added and the vial was placed in a heating block at 50 °C which was placed on top of a shaker (Thermolyne Maxi-Mix III). Aliquots (0.2 mL) were periodically taken and passed through a Celite 545plug (0.5 cm × 3 cm) and eluted with ether (4 mL). Gas chromatography was performed on the samples using the following method: initial temperature = 70 °C, initial time = 1 minute, ramp = 20 °C/min, final temperature = 250 °C, final time = 15 min.

### Catalytic reactions for Diels–Alder reactions

Stock solutions in acetonitrile were made of 3-(3-Furyl)pyridine, 4-(maleimidomethyl)pyridine and catalyst. Into a 15-dram screw-cap vial the substrates were weighted and each made up to a concentration of 9 × 10^–4^ M. The resulting mixture was stirred for 5 min before the catalyst (9.3 mg, 10 mol%) was added and the vial was then placed in a heating block at 50 °C which was placed on top of a shaker (Thermolyne Maxi-Mix III). After 48 h, aliquots were quickly passed through a Celite 545plug (0.5 cm × 3 cm) and then evaporated at low temperature. According to a previous research, the samples were analyzed by 400 MHz ^1^H NMR spectroscopy^31^. The relative amounts of the two adducts could be quantified by integration of a characteristic *exo* signal at 6.92 ppm and endo signals at both 6.84 and 6.94 ppm (Supplementary Fig. 29)^31^. The abovementioned procedure, carried out in duplicate on parallel samples for three times, gave reproducible exo:endo proportions.

## Supplementary information

Supplementary Information

Peer Review File

Source Data

## Data Availability

The authors declare that all the data supporting the findings of this study are available within the article (and Supplementary Information files), or available from the corresponding author on reasonable request. The source data underlying Figs. 1–5 are provided as a Source Data file.
