# Supplementary material for: Porous metal-metalloporphyrin gel as catalytic binding pocket for highly efficient synergistic catalysis
Source: Nat Commun. 2019 Apr 23;10:1913. doi: 10.1038/s41467-019-09881-9 (PMC6478723; doi:10.1038/s41467-019-09881-9)
Supplement: Supplementary file 1 — Supplementary Information [file 41467_2019_9881_MOESM1_ESM.pdf]

Supplementary Information

Porous Metal-Metalloporphyrin Gel as Catalytic Binding Pocket for Highly Efficient Synergistic Catalysis

Zhang et al.

Supplementary Figures

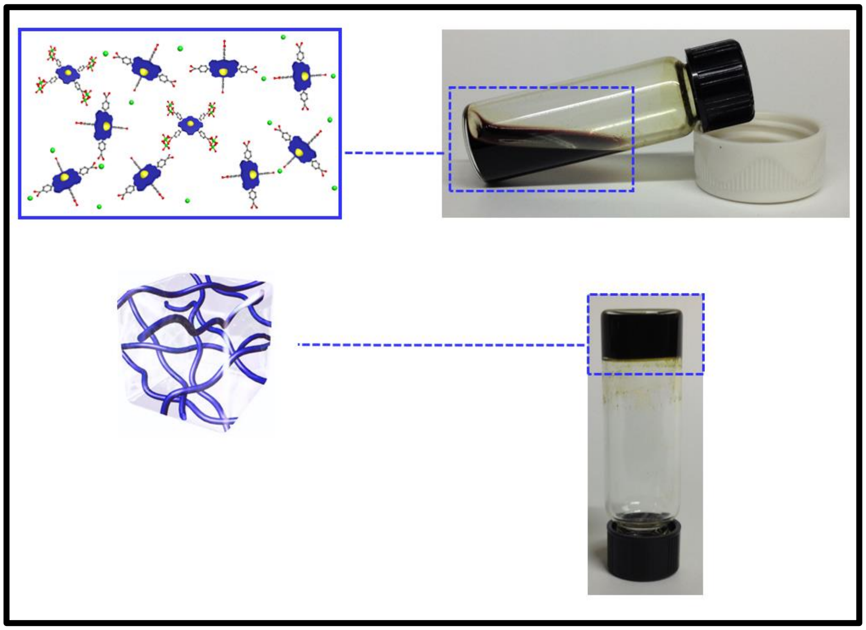

Supplementary Figure 1 | Optical image of ethanol solutions of Co-TCPP and $Al(NO_3)_3$ and metal-metalloporphyrin gel.

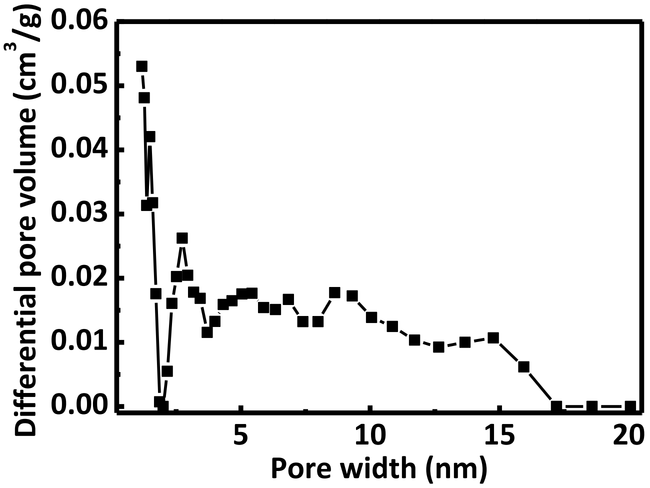

Supplementary Figure 2 | Pore size distribution analysis for Co-MMPG.

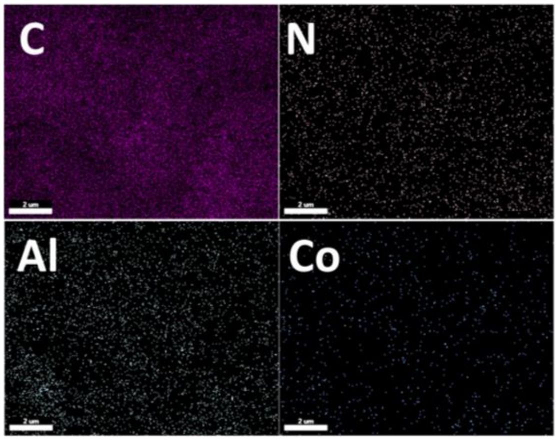

Supplementary Figure 3 | SEM-EDS analysis for Co-MMPG.

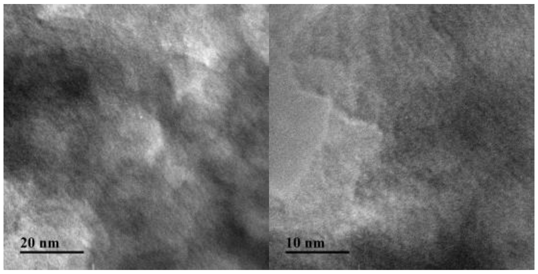

Supplementary Figure 4 | TEM analysis for Co-MMPG.

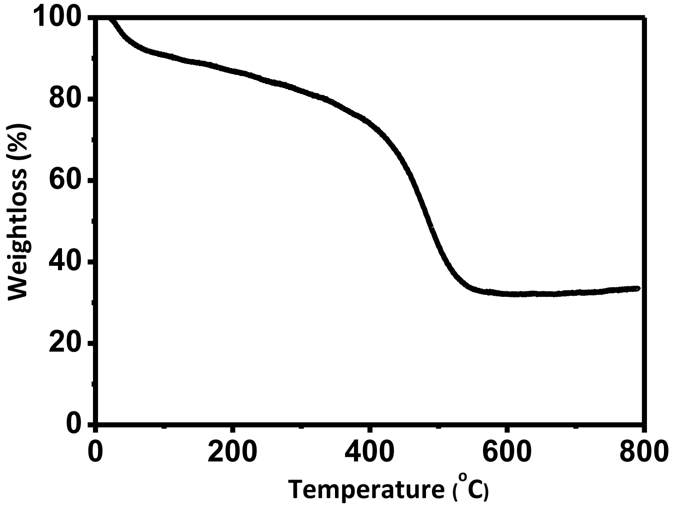

Supplementary Figure 5 | TG analysis for Co-MMPG.

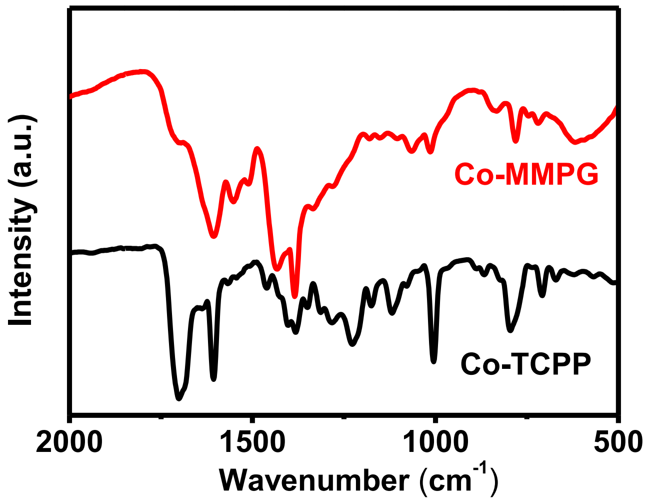

Supplementary Figure 6 | FT-IR analysis for Co-MMPG.

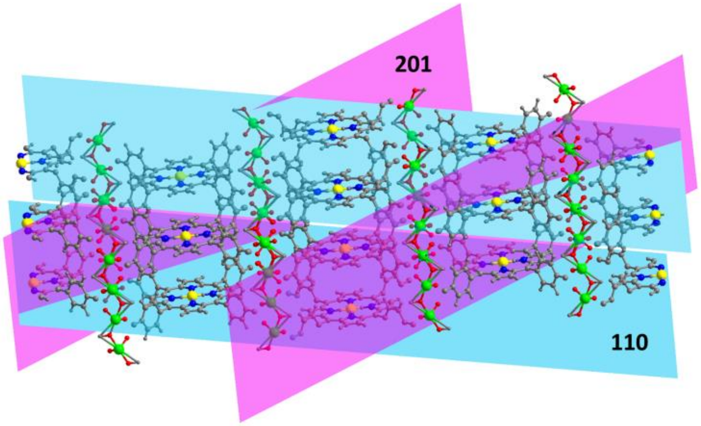

Supplementary Figure 7 | Finally expanded the concept of planes in Co-PMOF.

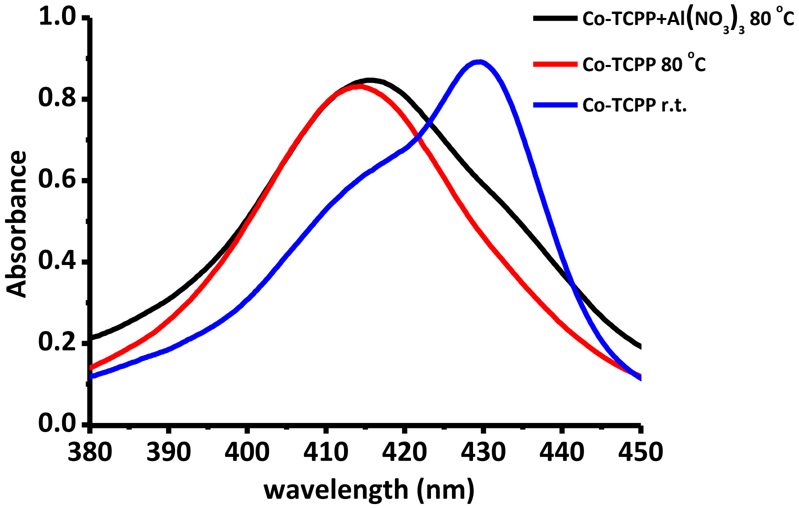

Supplementary Figure 8 | monitoring the formation of porphyrin-Al cluster in the ethanol solutions of Co-TCPP and $Al(NO_3)_3$ by UV-vis spectra. UV-vis absorption spectra comparing the ethanol solutions of Co-TCPP at room temperature, Co-TCPP at 80 °C, and Co-TCPP with $Al(NO_3)_3$ at 80 °C after 60 min.

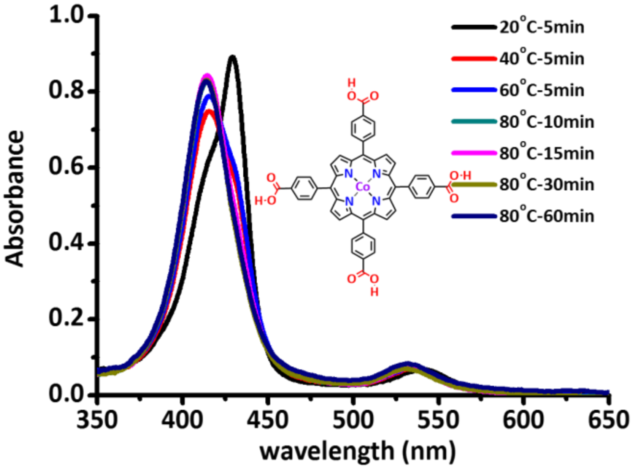

Supplementary Figure 9 | monitoring the formation of porphyrin-Al cluster in the ethanol solutions of Co-TCPP and Al(NO_3)$_3$ by UV-vis spectra. UV-vis spectra of an ethanol solutions containing 6 μM Co-TCPP monitored at different temperatures at different time intervals

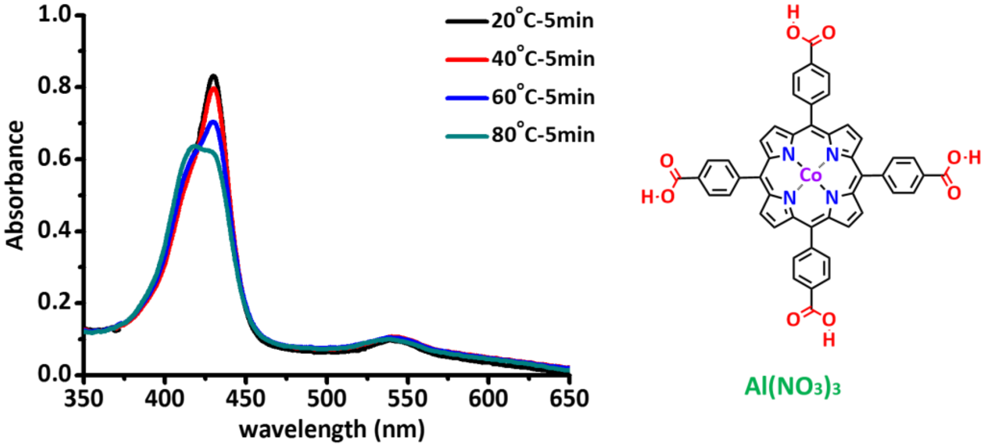

Supplementary Figure 10 | monitoring the formation of porphyrin-Al cluster in the ethanol solutions of Co-TCPP and Al(NO_3)$_3$ by UV-vis spectra. UV-vis of Co-TCPP (6 μM) with (12 μM) Al(NO_3)$_3$ at different temperatures

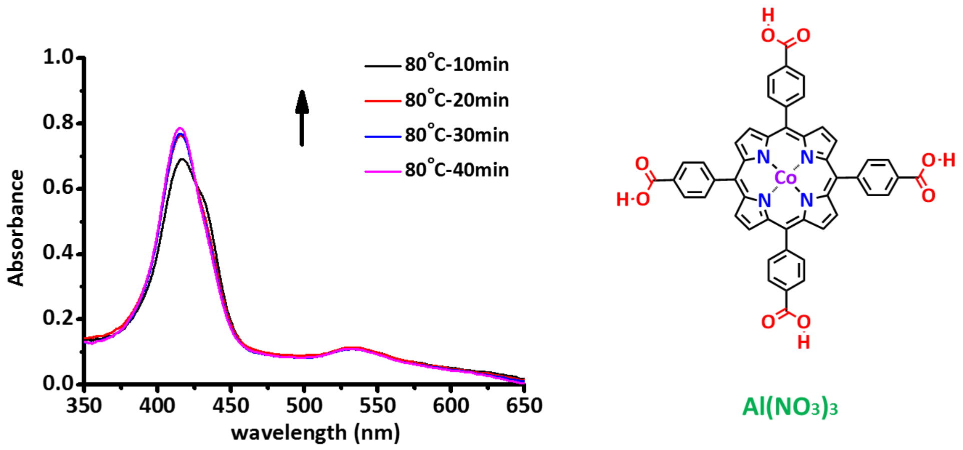

Supplementary Figure 11 | monitoring the formation of porphyrin-Al cluster in the ethanol solutions of Co-TCPP and $Al(NO_3)_3$ by UV-vis spectra. UV-vis spectra of ethanol solutions at 80 °C and maintain 40 min in the presence of Co-TCPP (6 μM) with (12 μM) $Al(NO_3)_3$

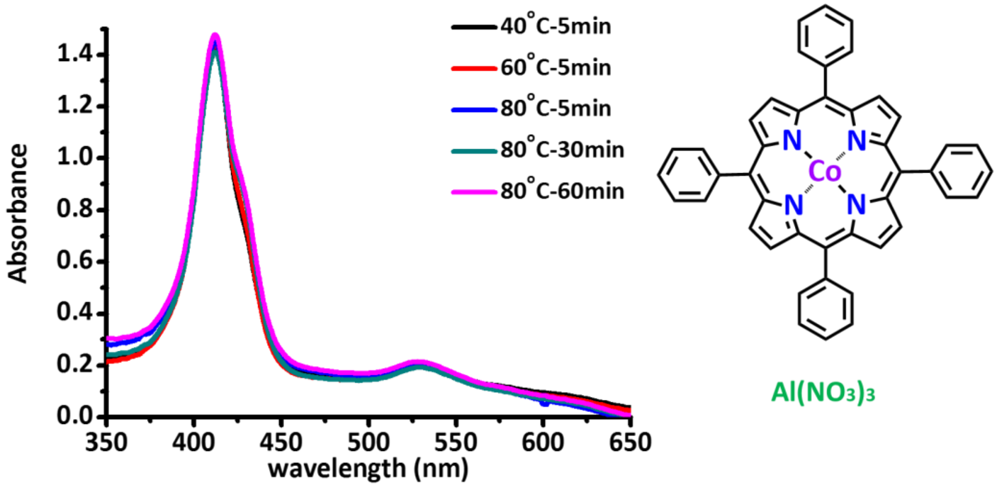

Supplementary Figure 12 | monitoring the formation of porphyrin-Al cluster in the ethanol solutions of Co-TCPP and $Al(NO_3)_3$ by UV-vis spectra. The UV-vis of 6 μM Co-TPP with (12 μM) $Al(NO_3)_3$ at different temperatures and time intervals.

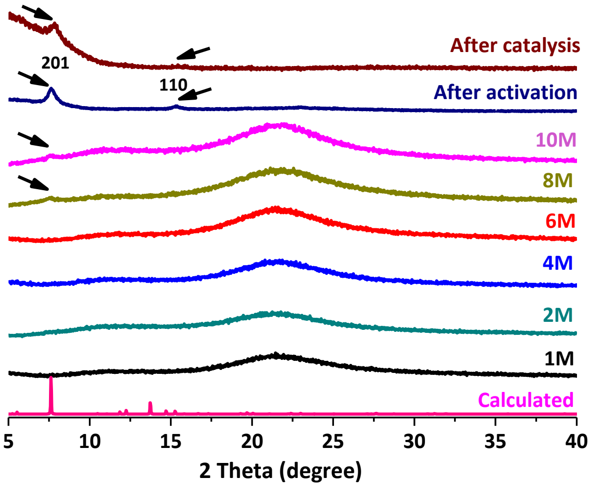

Supplementary Figure 13 | PXRD patterns for Co-MMPG in various concentration and calculated ones based on single X-ray data[1].

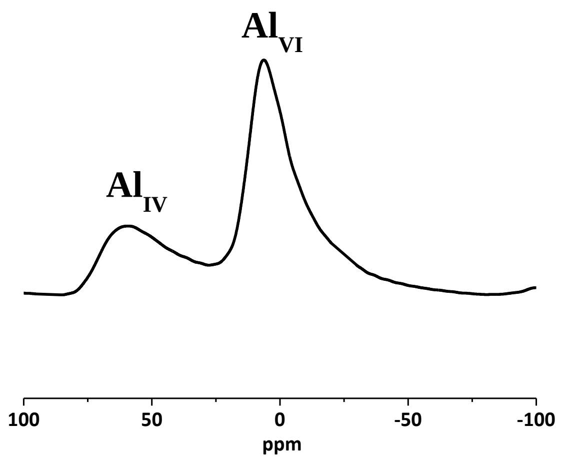

Supplementary Figure 14 | ^{27}Al MAS NMR spectrum of Co-MMPG.

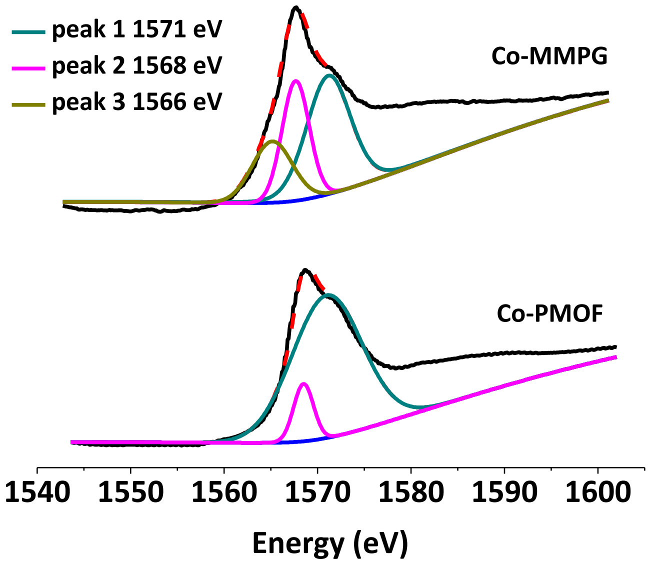

Supplementary Figure 15 | Al K-edge XANES spectra for Co-PMOF and Co-MMPG, peak maximum at 1571 eV, 1568 eV and 1566 eV.

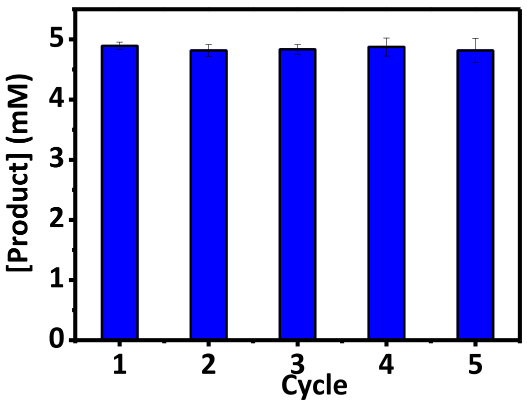

Supplementary Figure 16 | Reusability of the Co-MMPG in five reaction runs. The S.E.M. represents the standard deviation from the repeated experiment after three times.

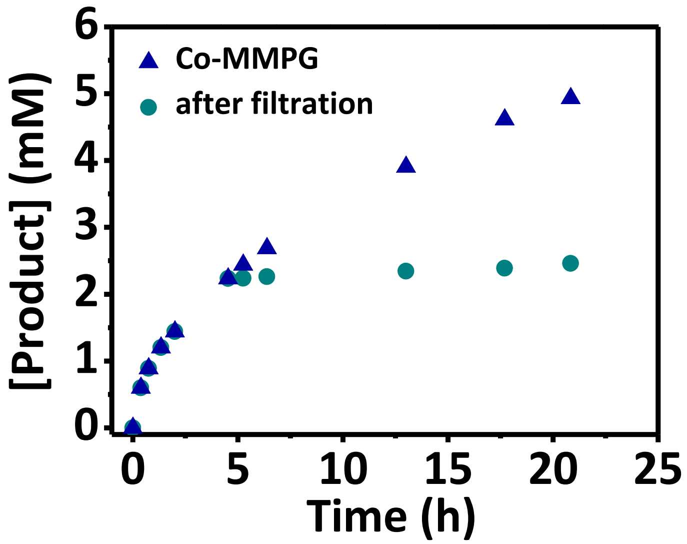

Supplementary Figure 17 | Kinetic profile of producing 3-acetoxymethylpyridines catalyzed by Co-MMPG before or after a hot filtration.

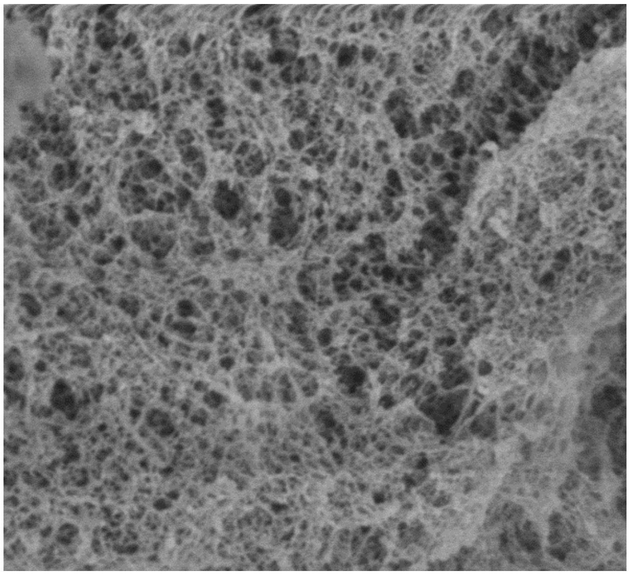

Supplementary Figure 18 | SEM of Co-MMPG after reused

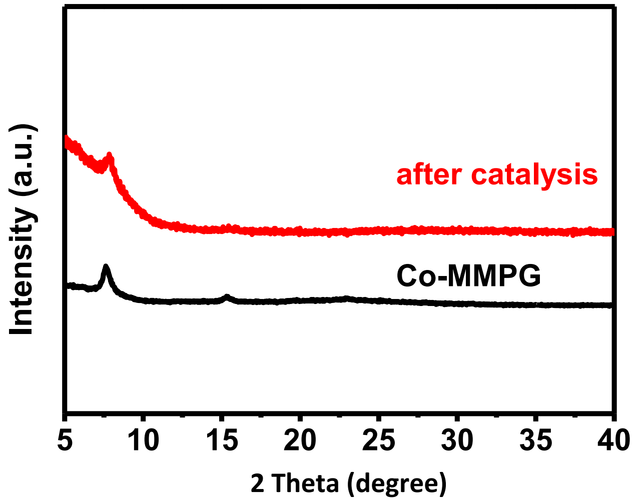

Supplementary Figure 19 | PXRD patterns for fresh and recycled Co-MMPG

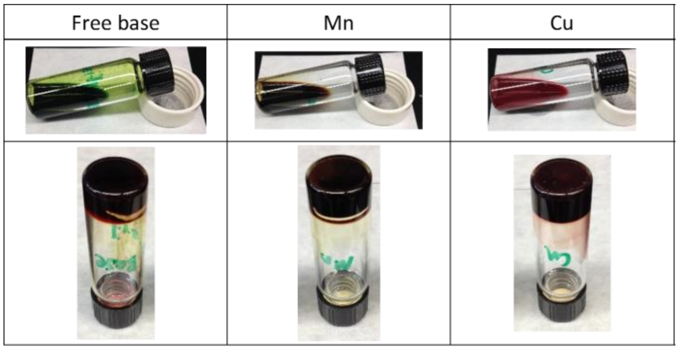

Supplementary Figure 20 | Optical image of ethanol solutions of TCPP and $Al(NO_3)_3$, Mn-TCPP and $Al(NO_3)_3$, Cu-TCPP and $Al(NO_3)_3$ and their metal-metalloporphyrin gel.

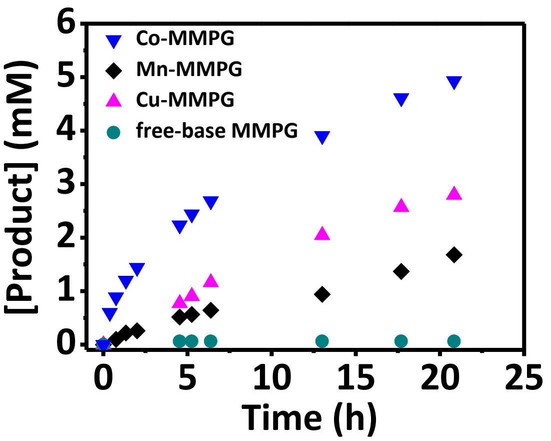

Supplementary Figure 21 | Catalytic property tests. (a) Plot of product conversion versus time, showing the initial production of the various isomers catalyzed by various gel catalysts;

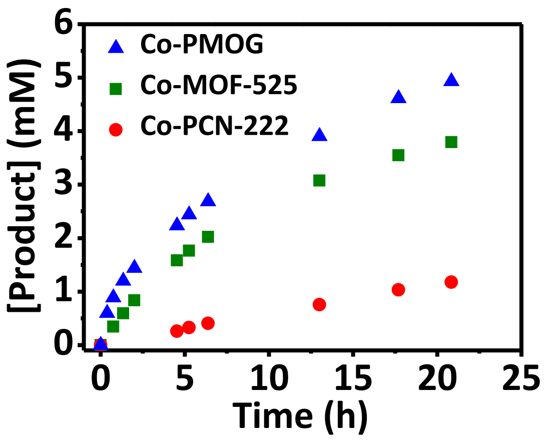

Supplementary Figure 22 | Catalytic property tests. Plot of product conversion versus time, showing the initial production of the various isomers catalyzed by various porphyrin MOF catalysts.

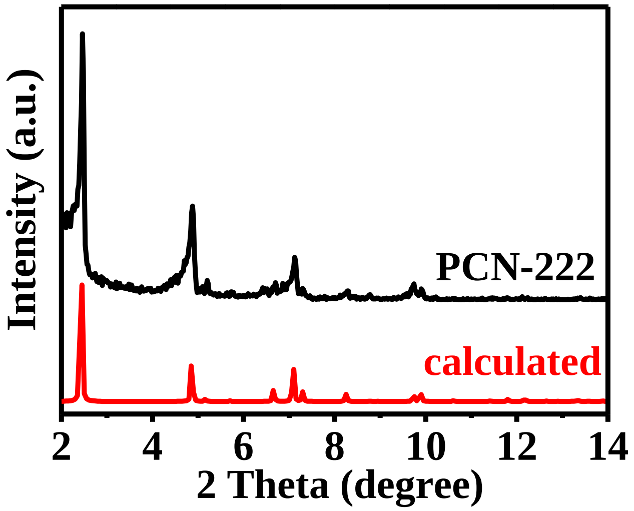

Supplementary Figure 23 | PXRD patterns for PCN-222 and calculated data based on reported single X-ray data.

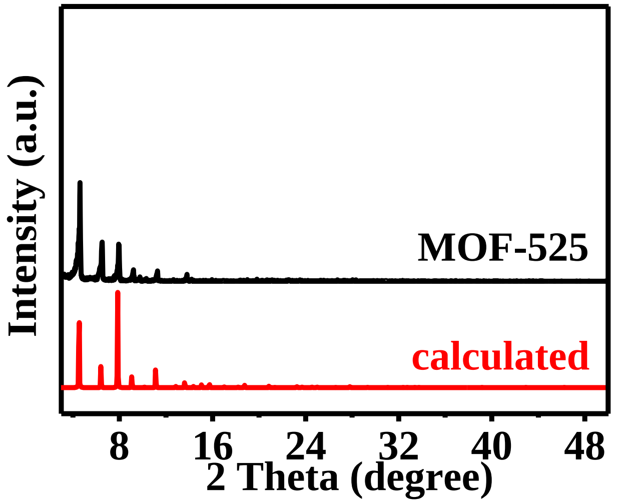

Supplementary Figure 24 | PXRD patterns for MOF-525 and calculated data based on reported single X-ray data.

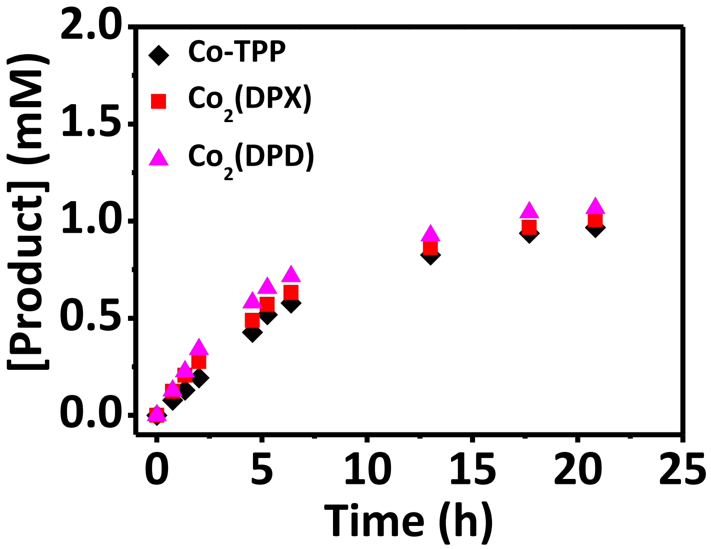

Supplementary Figure 25 | Catalytic property tests. Plot of product conversion versus time, showing the initial production of the various isomers catalyzed by various porphyrin dimers.

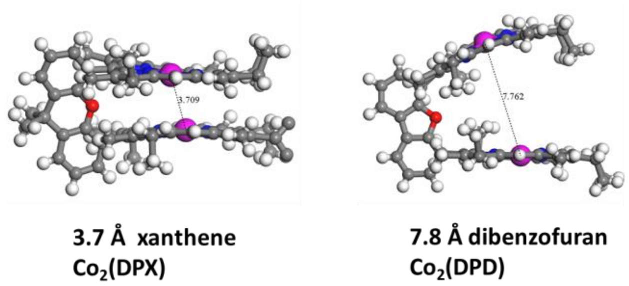

Supplementary Figure 26 | Crystal structure information about Co-Co distance of two porphyrin dimers.

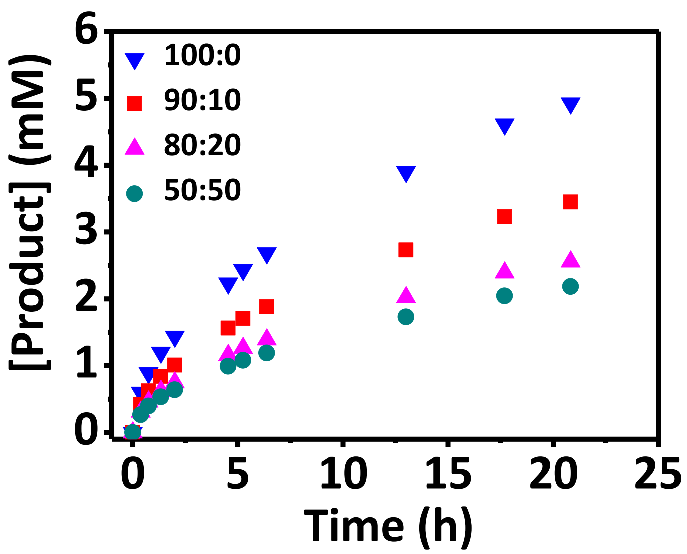

Supplementary Figure 27 | Catalytic property tests. Plot of product conversion versus time, showing the initial production of the various isomers catalyzed by Co-MMPG with various Co content (100 %, 90 %, 80 % and 50 %).

Supplementary Table 1 | Comparison of the catalytic activity of relevant porphyrin catalysts for the acyl-transfer reaction.

Catalyst	Initial rate constant	Concentration of N-acetylimidazole	Concentration of metal sites (wt %)	Temperature	Journal
MOF-902	0.33 mM/h	6 mM	9.82 % [a] (5 % [b])	60 °C	J. Am. Chem. Soc. 138, 14449-14457 (2016)
MOF-525	0.67 mM/h	6 mM	8 % [a] (6 % [b])	60 °C	J. Am. Chem. Soc. 138, 14449-14457 (2016)
PCN-222	0.07 mM/h	6 mM	8.71 % [a] (5 % [b])	60 °C	J. Am. Chem. Soc. 138, 14449-14457 (2016)
Microporous Metalloporphyrin-Containing Framework	0.3 mM/h	20 mM	Not applicable	60 °C	Angew. Chem. Int. Ed. 55, 6013-6017 (2016)
Cyclic Porphyrin Trimer (trimer 1 with $CH_2CH_2COOCH_3$ as R group)	0.18 mM/h	9 mM	6 % [b]	70 °C	J. Am. Chem. Soc., 116, 3141-3142 (1994)
ZnPO-MOF	0.6 mM/h	6 mM	3 % [b]	60 °C	J. Am. Chem. Soc., 131, 4204-4205 (2009)
Monomeric Co-TPP	0.13 mM/h	6 mM	10 % [b]	50 °C	This work
Co-MMPG	0.70 mM/h	6 mM	1 % [a]	50 °C	This work
Co_2(DPX)	0.15 mM/h	6 mM	9 % [b]	50 °C	This work
Co_2(DPD)	0.17 mM/h	6 mM	10 % [b]	50 °C	This work
Co-PMOF	0.10 mM/h	6 mM	5.2 % [b]	50 °C	This work
Co-MOF-525	0.46 mM/h	6 mM	5.8 % [b]	50 °C	This work

[a] Obtained from EDS experiments

[b] Obtained from crystal structures

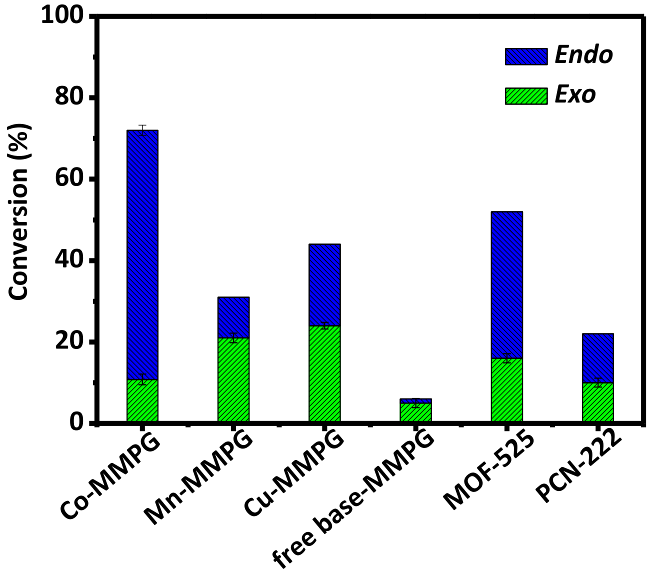

Supplementary Figure 28 | Catalytic Performance for Diels-Alder reactions. Conversion and selectivity of *exo* and *endo* reactions in the presence of various catalysts. The S.E.M. represents the standard deviation from the repeated experiment after three times.

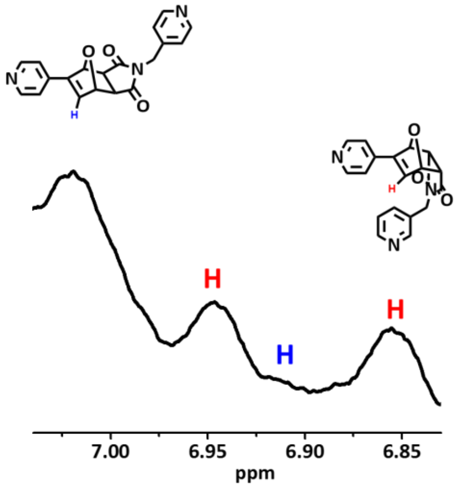

Supplementary Figure 29 | ^{1}H-NMR spectra of *exo* and *endo* adducts in DMSO-d_6 at room temperature. The relative amounts of the two adducts could be quantified (5%) by integration of a characteristic *exo* signal at 6.92 ppm and *endo* signals at 6.85 and 6.94 ppm.[2]

Supplementary Reference

1. Fateeva, A. et al. A water-stable porphyrin-based metal-organic framework active for visible-light photocatalysis. *Angew. Chem. Int. Ed.* **51**, 7440-7444 (2012).
2. Clyde-Watson, Z. *et al.* Reversing the stereochemistry of a Diels-Alder reaction: use of metalloporphyrin oligomers to control transition state stability. *New J. Chem.* **22**, 493-502 (1998).
