# Supplementary material for: Porous metal-metalloporphyrin gel as catalytic binding pocket for highly efficient synergistic catalysis
Source: Nat Commun. 2019 Apr 23;10:1913. doi: 10.1038/s41467-019-09881-9 (PMC6478723; doi:10.1038/s41467-019-09881-9)
Supplement: Supplementary file 2 — Peer Review File [file 41467_2019_9881_MOESM2_ESM.pdf]

Reviewers' comments:

Reviewer #1 (Remarks to the Author):

This study presents the first aerogel capable of enzyme-mimic synergistic catalysis by fabricating a metal-metalloporphyrin gel catalyst with hierarchical porosity. The cooperativity between two Co(II) sites within the defined nanospace pocket facilitates the binding of different substrates with a favorable geometry thereby rendering Co-MMPG with excellent performance in the context of synergistic catalysis, especially for the kinetic control stereoselective reactions. Its structure has been well elucidated through the combined studies of solid-state NMR and X-ray absorption near-edge structure (XANES). Further examination catalyzed by other catalysts reveals the superior performance of Co-MMPG particularly in comparison with the rigid MOF counterparts of PCN-222 and MOF-525, highlighting the importance of the local structure of pocket with suitably oriented “neighboring active sites”. Its performance promoted a joint structural and catalytic study to rationalize the origin of this behavior. Conceptually, this work suggests a new approach for enzyme mimic design of highly efficient heterogeneous catalyst to carry out chemo/stereo-selective chemistry. Moreover, it also advances metal organic gels as a new platform for heterogeneous catalysis, which will attract increasing attention in the community. This reviewer urges the publication of this work in Nature Communications after minor revisions with the following comments addressed.

1. Some routine characterizations of the Co-MMPG material should be conducted, such as IR and TGA,

2. In the main text, the authors stated no detectable leaching of active site and cobalt ions in the reaction solution were observed after removal of Co-MMPG by filtration. Also, the authors stated Co-MMPG can be reused for five cycles without significant drop in its catalytic activity (Supplementary Fig. 14). It is suggested a hot filtration test be added to further prove the heterogeneous catalysis nature.

3. Along this line, textual characterization of C-MMPG catalyst after reactions is suggested to be conducted, such as SEM and PXRD.

Reviewer #2 (Remarks to the Author):

“Porous Metal-Metalloporphyrin Gel as Catalytic Binding Pocket for Highly Efficient Synergistic Catalysis” by Prof Ma reports a fibrous gel prepared from Co(II)-metalated tetratopic porphyrin linkers acting as coordination-mediated Lewis acid center for catalysis of acyl-transfer and Diels-Alder reactions. The authors utilized various techniques to glean on the structural component of the gel materials.

Following are the points that the editor office requested to evaluate this manuscript on.

1. What are the major claims of the paper?
Synergistic catalysis stemming from a flexible binding pocket formed from the co-facial arrangement of the metalloporphyrin rings in the reported aerogel materials.

2. Are they novel and will they be of interest to others in the community and the wider field?

The aerogel materials may be a competitive (relative to MOF based system) new composition where flexibility is claimed to be a high point. But flexibility would stand for poor selectivity –a wider range of

‘non-fitting’ substrate can be accommodated?
Also, based on the work presented here, it is not clear how easy to control the dimensionality of the binding pocket compared to structurally well-established MOF system (such as those systematic variations shown in ref #8). It is also not sure how the concentration of the catalytically active center can be varied as the performance of these heterogeneous systems would depend on the local concentration of the active site and substrate matching of the binding pocket (synergy).

While the authors, with various techniques, tried to establish the structure of the gel material as such the structural information such as the dimension of the binding pocket (e.g. Co-Co distance) is missing. Is this suitable to host and stabilize the intermediate of the reaction of 3-PC and N-acyl-imidazole? As described in ref 8, how this dimension compared to the MOF-525?

Without knowing these parameters, choice of the control catalysts would be vague. For example, the other Co-PMOF system reported by Rosseinsky (ref 40) has very closely placed porphyrins in Al-TCPP MOF. This close packing leads to inter porphyrin distance much shorter than the intermediate of the reaction of 3-PC and N-acyl-imidazole. So how this would be a good control? Perhaps the better control would be MOF-525(Co).

It also should be compared to a cofacial porphyrin dimer that Anson, Nocera, Coleman studied with xanthene, anthracene, dibenzofuran spacers? Monomeric Co-TPP is a control that does not have concerted tailor-made binding sites.

3. If the conclusions are not original, it would be helpful if you could provide relevant references.

While the acyl-transfer reaction with PC studied here provide tools to establish the effect of specific structural arrangement to host/bind both the substrates, the structure-efficiency was thoroughly established in ref 8. How this rate constant compared with those?

4. Is the work convincing, and if not, what further evidence would be required to strengthen the conclusions?

a. The results are partially convincing. The rate constants need to be extracted and compared with the relevant MOF system.
b. The NMR based proposed mechanism (electronic effect of the coordinated PC; Fig 4) is not convincing as the –OH group is not directly attached to the pyridine. Not sure what the authors wanted to convey in Fig4b. if it is various resonance structures drawn with the one-sided arrow it is not correct; if it represents various TS/intermediates then not sure what it means.
c. Scheme 1 does not clearly convey what is the backbone of the fiber if it involved an Al-O based polymer chain.
d. Not clear what is the H-bond system the authors mentioned several times to describe the structure.
e. It is also not clear what would cause the change in 415 nm Soret band by altering ‘aggregation’.
f. ICP data suggest 1.0wt% Co content, so what is the point? Can these be changed in a controlled manner? How would the catalytic efficiency vary with that?

5. On a more subjective note, do you feel that the paper will influence thinking in the field?
Not in the current form. The true potential of a new composition, such as MMPG, must establish a better and/or easier control of the structure-property to tie the performance (power to control the mechanism/reaction-pathway/ choice of substrate/stereogenic control of the product) to influence thinking in the field.
On the contrary, the endo/exo DA reaction would be more interesting to explore/develop this work.

Minor point:
the figures are not very clear/readable.

Dr. Shengqian Ma
Professor

Department of Chemistry
University of South Florida
4202 E. Fowler Avenue, CHM 205A
Tampa, FL 33620

Phone: 1-813-974-5217
Fax: 1-813-974-3203
E-mail: sqma@usf.edu
Website: http://sqma.myweb.usf.edu/

Date: February 20, 2019

We greatly appreciate the constructive comments and suggestions from all reviewers, and we have revised the manuscript accordingly as detailed in the responses below. The corresponding changes have been highlighted in yellow in the main text and Supplementary Information.

Reviewer #1:

This study presents the first aerogel capable of enzyme-mimic synergistic catalysis by fabricating a metal-metalloporphyrin gel catalyst with hierarchical porosity. The cooperativity between two Co(II) sites within the defined nanospace pocket facilitates the binding of different substrates with a favorable geometry thereby rendering Co-MMPG with excellent performance in the context of synergistic catalysis, especially for the kinetic control stereoselective reactions. Its structure has been well elucidated through the combined studies of solid-state NMR and X-ray absorption near-edge structure (XANES). Further examination catalyzed by other catalysts reveals the superior performance of Co-MMPG particularly in comparison with the rigid MOF counterparts of PCN-222 and MOF-525, highlighting the importance of the local structure of pocket with suitably oriented "neighboring active sites". Its performance promoted a joint structural and catalytic study to rationalize the origin of this behavior. Conceptually, this work suggests a new approach for enzyme mimic design of highly efficient heterogeneous catalyst to carry out chemo/stereo-selective chemistry. Moreover, it also advances metal organic gels as a new platform for heterogeneous catalysis, which will attract increasing attention in the community. This reviewer urges the publication of this work in Nature Communications after minor revisions with the following comments addressed.
Response: We thank the reviewer for taking the time to review our manuscript and appreciate the high comments and support for our work from the reviewer.

Comment 1: Some routine characterizations of the Co-MMPG material should be conducted, such as IR and TGA.
Response: We thank the reviewer for the valuable suggestion. We have supplied some routine characterizations of Co-MMPG into Supplementary Information, such IR and TGA. Co-MMPG is stable up to 380 ºC and its decomposition starts at or after 380 ºC (Supplementary Fig. 5). For Co-TCPP, the absorption bands centered at 1704 cm^{-1} were observed, which can be attributed to the non-coordinated COOH groups. For Co-MMPG, the strength of COOH groups greatly decreased, which indicated that COOH coordinates with Al after the gel formation (Supplementary Fig. 6).

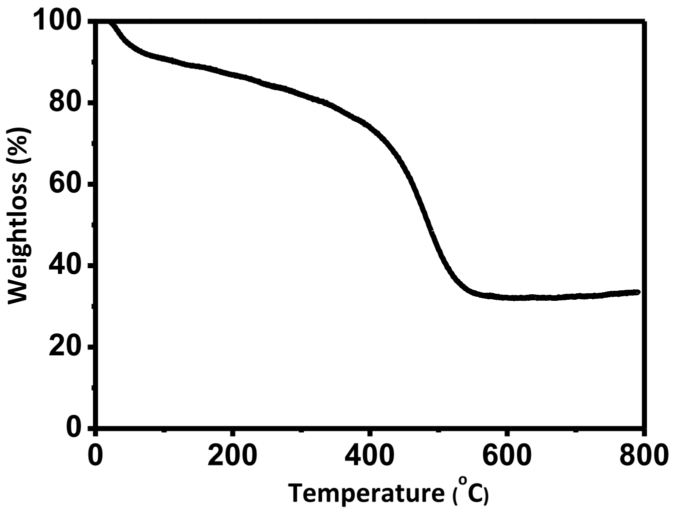

Supplementary Figure 5 | TG analysis for Co-MMPG.

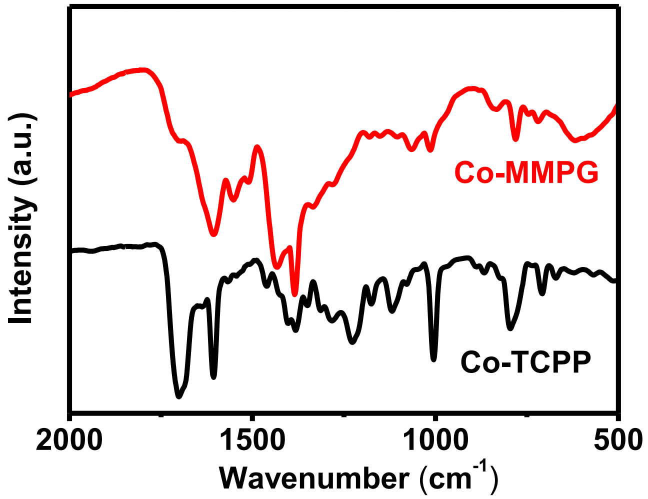

Supplementary Figure 6 | FT-IR analysis for Co-MMPG.

Comment 2: In the main text, the authors stated no detectable leaching of active site and cobalt ions in the reaction solution were observed after removal of Co-MMPG by filtration. Also, the authors stated Co-MMPG can be reused for five cycles without significant drop in its catalytic activity (Supplementary Fig. 14). It is suggested a hot filtration test be added to further prove the heterogeneous catalysis nature.

Response: We thank the reviewer for the comment. A hot filtration test has been included in the Supplementary Information to investigate the leaching of the catalyst during reaction. There was no obvious increase in conversion after a hot filtration of Co-MMPG (Supplementary Fig. 17). This indicates that acyl-transfer reactions catalyzed by Co-MMPG is a heterogeneous catalysis.

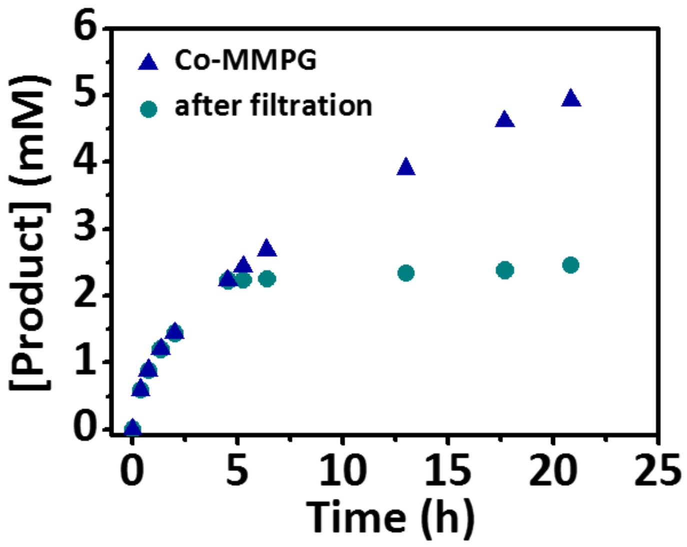

Supplementary Figure 17 | Kinetic profile of producing 3-acetoxymethylpyridines catalyzed by Co-MMPG before or after a hot filtration.

Comment 3: Along this line, textual characterization of Co-MMPG catalyst after reactions is suggested to be conducted, such as SEM and PXRD.

Response: We appreciate the reviewer for the valuable comments. Per the reviewer's suggestion, the SEM and PXRD have been carefully investigated. No morphological changes of Co-MMPG were observed (Supplementary Fig. 18). The PXRD peak positions were the same in both but the relative intensity of the peaks were slightly different for fresh and recycled Co-MMPG (Supplementary Fig. 19).

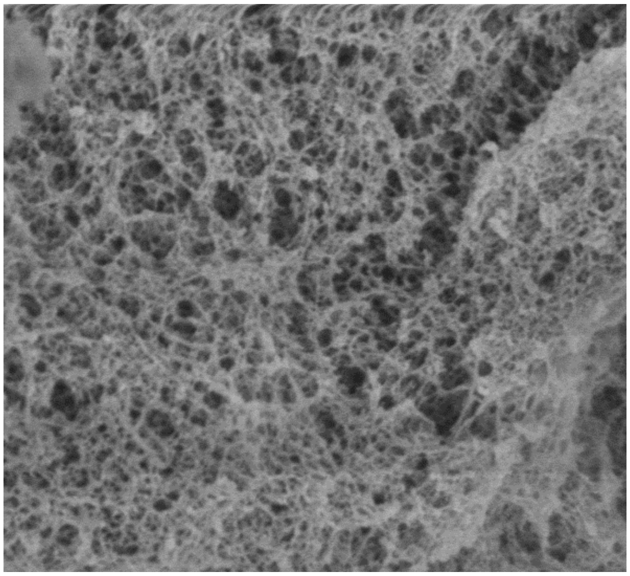

Supplementary Figure 18 | SEM of Co-MMPG after reused.

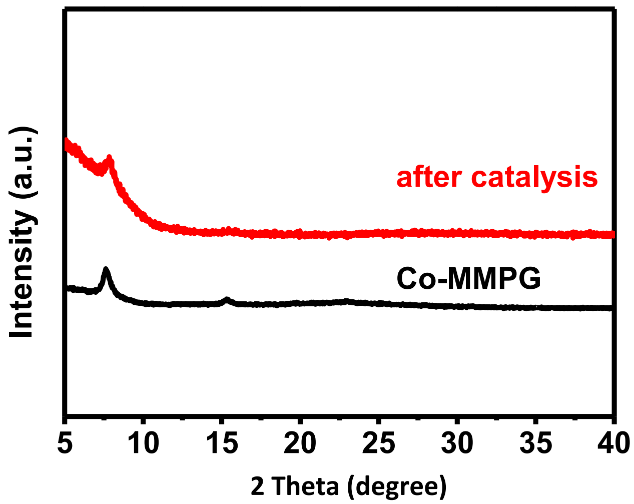

Supplementary Figure 19 | PXRD patterns for fresh and recycled Co-MMPG.

Reviewer #2:

"Porous Metal-Metalloporphyrin Gel as Catalytic Binding Pocket for Highly Efficient Synergistic Catalysis" by Prof Ma reports a fibrous gel prepared from Co(II)-metalated tetratopic porphyrin linkers acting as coordination-mediated Lewis acid center for catalysis of acyl-transfer and Diels-Alder reactions. The authors utilized various techniques to glean on the structural component of the gel materials.

Response: We thank the reviewer for taking the time to review our manuscript and valuable comments. Per the reviewer's suggestion, we further demonstrate the major claims of our paper and analyze the structure-performance relationships. The structure-efficiency has been thoroughly established *via* the comparison with more control experiments, like the catalytic activity of homogeneous porphyrin monomer, dimer, trimer, microporous metalloporphyrin-containing polymer and MOFs.

Following are the points that the editor office requested to evaluate this manuscript on.

Comment 1: What are the major claims of the paper? Synergistic catalysis stemming from a flexible binding pocket formed from the co-facial arrangement of the metalloporphyrin rings in the reported aerogel materials.

Response: We are thankful to the reviewer for the comments. As the reviewer mentioned, the major claims of this paper is "Synergistic catalysis stemming from a flexible binding pocket formed from the co-facial arrangement of the metalloporphyrin rings in the aerogel materials". Synergistic catalysis is a synthetic strategy wherein both the nucleophile and the electrophile are simultaneously activated by two separate and distinct catalysts to afford a single chemical transformation. In this work, this powerful catalysis strategy leads to several benefits, specifically synergistic catalysis can improve the efficiency of existing transformations, and create or improve catalytic selectivity where stereo-control was previously absent or

challenging. Metal-metalloporphyrin gel was selected to combine acyl-transfer with Diels-Alder reactions *via* a critical thinking process based on synergistic catalysis.

Comment 2: Are they novel and will they be of interest to others in the community and the wider field? The aerogel materials may be a competitive (relative to MOF based system) new composition where flexibility is claimed to be a high point. But flexibility would stand for poor selectivity-a wider range of 'non-fitting' substrate can be accommodated?

Also, based on the work presented here, it is not clear how easy to control the dimensionality of the binding pocket compared to structurally well-established MOF system (such as those systematic variations shown in ref #8). It is also not sure how the concentration of the catalytically active center can be varied as the performance of these heterogeneous systems would depend on the local concentration of the active site and substrate matching of the binding pocket (synergy). While the authors, with various techniques, tried to establish the structure of the gel material as such the structural information such as the dimension of the binding pocket (e.g. Co-Co distance) is missing. Is this suitable to host and stabilize the intermediate of the reaction of 3-PC and N-acyl-imidazole? As described in ref 8, how this dimension compared to the MOF-525?

Response: We are thankful to the reviewer for the comments.

It is well-known that an enzyme is able to perform highly efficient catalysis because of its structural flexibility or conformational adaptability. For enzymatic catalysis, it is essential to be flexible but not too flexible, as well as rigid but not too rigid (*Nature,* 2019*, 565,* 213). Specifically, the protein must be rigid enough to maintain the required structure but flexible enough to permit atomic movements as the reaction proceeds. Therefore, we believe that the catalysis of porphyrin MOFs will be boosted if the flexibility can be introduced into frameworks. As demonstrated in our work, metal organic gels exhibited superior catalysis performance in terms of both activity and selectivity, which can be primarily attributable to the synergistic effect of two Co(II) binding sites within the porphyrin binding pocket. This can be ascribed to the inherent correlation between MOFs and MOGs with comparable bonding connectivity and conformational flexibility.

We propose herein the construction of hierarchically porous MOGs that can be combined to show similar coordination found in MOFs (*Adv. Mater. 2014, 26, 2072, Angew. Chem. Int. Ed. 2009, 48, 2325*), capable of mimicking the binding pocket/active center of enzyme yet show greater conformational flexibility similar to organic gels. Although it is extremely difficult to get the precise Co-Co distance from a structural point of view due to the lack of crystallinity for gel, this distance range can be estimated based on the MOF structure. As shown in Fig. 1c, PXRD studies showed that Co-MMPG retained its structural integrity after activation; the broader peaks towards lower 2 theta values are due to the aggregates with conformational flexibility caused by twisting Al-O-Al cluster. Interestingly, it has been reported that the Al-O-Al bond angel distribution ranges from 80 to 180 (*J. Chem. Phys. 2013, 139, 044507*). We then carefully estimated the Co-Co distance based on the MOF structure. The Al-O-Al bond angel distribution leads to a distance range of Co-Co distance from 6.8 Å to 16.7 Å (as shown below).

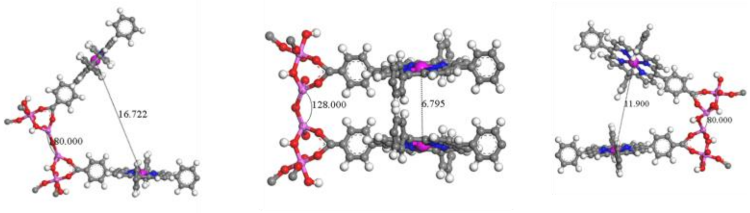

The metal-metal distances of all porphyrin pairs in MOF-525 (13.6 Å), NU-902 (10.5 Å) and PCN-222 (9.9 Å) are moderately to significantly larger than the N_{NAI}-N_{PC} distances (6.7 Å), which would rule out all porphyrin pairs (*J. Am. Chem. Soc. 2016, 138, 14449*). Thus, it follows that the facilitation of the transition state due to complementary coordination may be aided by the "breathing" of the MOF bringing NAI and PC closer together and/or that some level of strain is permissible and necessary to form the transition state (please also note that during anchoring N can only come as close to metal site as the N-metal bond distance allows). In relative terms, the strain energy for the anchored intermediate is too high in the porphyrin pairs of parallel type or with too long distance (> 18.2 Å). Therefore, on the basis of Co-Co distance, the rate enhancement due to preconcentration effects arises when NAI and PC diffuse into the MOGs and bind to the porphyrins *via* N_{NAI}-Co and N_{PC}-Co interactions. The binding populates the porphyrins with NAI and PC, resulting in a large number of molecules confined in a small volume, making it more probable for them to encounter each other and react. More importantly, a relationship model was successfully established, which could be used to predict and regulate the geometrical dimensions of MOGs. From a purely practical point of view, a convenient preparation is realized, leading to that specific microstructure can be prepared rapidly and accurately instead of suffering from long-time experimental synthesis in the future.

Without knowing these parameters, choice of the control catalysts would be vague. For example, the other Co-PMOF system reported by Rosseinsky (ref 40) has very closely placed porphyrins in Al-TCPP MOF. This close packing leads to inter porphyrin distance much shorter than the intermediate of the reaction of 3-PC and N-acyl-imidazole. So how this would be a good control? Perhaps the better control would be MOF-525(Co).

Response: We are thankful to the reviewer for the comments. The highest initial rate for 3-PC with MOF-525 is about double that of the reported NU-902 (*J. Am. Chem. Soc. 2016, 138, 14449*). It can be deduced that Co-Co distance of no less than 10 Å together with favorable orientation and proximal positioning of NAI and PC by metal sites favors the rate enhancement. Further examination of 3-AMP formation catalyzed by other Co(II)-based porphyrin MOF catalysts reveals the superior performance of Co-MMPG in comparison with PCN-222 and MOF-525 (Supplementary Fig. 22), highlighting the importance of the local pocket structure with suitably oriented "neighboring active sites".

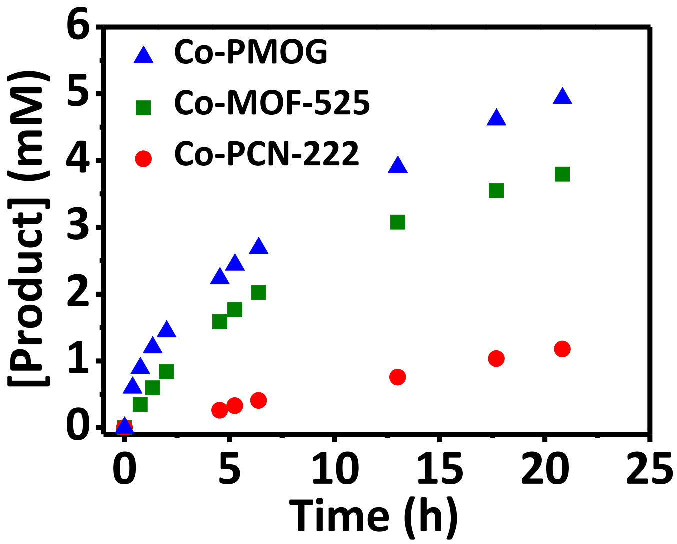

Supplementary Figure 22 | Catalytic property tests. Plot of product conversion versus time, showing the initial production of the various isomers catalyzed by various porphyrin MOF catalysts.

It also should be compared to a cofacial porphyrin dimer that Anson, Nocera, Coleman studied with xanthene, anthracene, dibenzofuran spacers? Monomeric Co-TPP is a control that does not have concerted tailor-made binding sites.

Response: We are thankful to the reviewer for the comments. This is a great suggestion! A homologous family of di-cobalt(II) Pacman porphyrins anchored by xanthene [Co_2(DPX)] and dibenzofuran [Co_2(DPD)] have been synthesized (*Inorg. Chem. 2002, 41, 3102*). We then analyzed the dimension of the binding pocket inside Co_2(DPX) and Co_2(DPD), especially the Co-Co distance. The Co-Co distances of all porphyrin dimers in Co_2(DPX) (3.7 Å) and Co_2(DPD) (7.8 Å) are significantly shorter than the necessary distances for the intermediate. Therefore, the strain energy for the anchored intermediate should be very high inside Co_2(DPX) and Co_2(DPD). Compared with the results of Co-TPP, the di-Co(II) Pacman porphyrins anchored by xanthene [Co_2(DPX)] and dibenzofuran [Co_2(DPD)] did not display enhanced catalytic performance (Supplementary Fig. 25), further highlighting the importance of the local structure of pocket with suitably oriented "neighboring active sites".

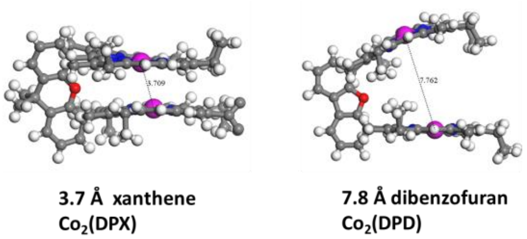

Supplementary Figure 26 | Crystal structure information about Co-Co distance of two porphyrin dimers.

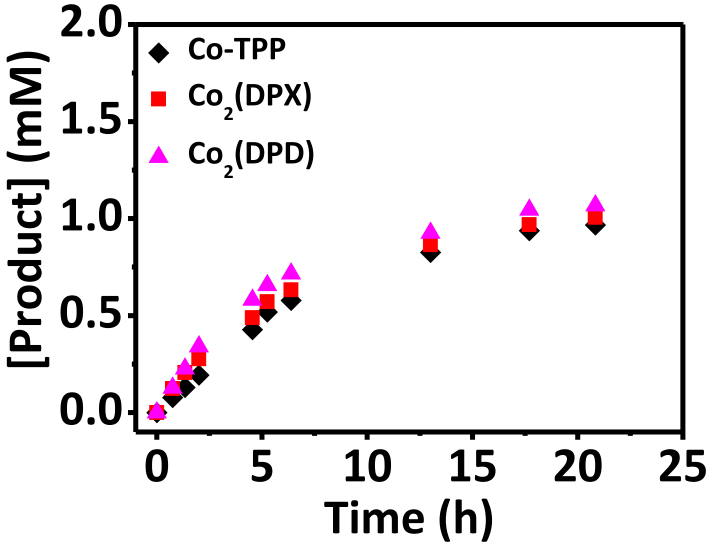

Supplementary Figure 25 | Catalytic property tests. Plot of product conversion versus time, showing the initial production of the various isomers catalyzed by various porphyrin dimers.

3. If the conclusions are not original, it would be helpful if you could provide relevant references. While the acyl-transfer reaction with PC studied here provide tools to establish the effect of specific structural arrangement to host/bind both the substrates, the structure-efficiency was thoroughly established in ref 8. How this rate constant compared with those?

Response: We are thankful to the reviewer for the criticism. An inferior activity has been observed for the Co(II)-based porphyrin MOF catalysts of PCN-222, MOF-525, MOF-902 and other previously reported catalysts based on porphyrin in comparison with Co-MMPG (Supplementary Table 1).

Supplementary Table 1 | Comparison of the catalytic activity of relevant porphyrin catalysts for the acyl-transfer reaction.

Catalyst	Rate constant	Concentration	Temperature	Journal
MOF-902	0.33 mM/h	6 mM	60 ºC	J. Am. Chem. Soc. 138, 14449-14457 (2016)
MOF-525	0.67 mM/h	6 mM	60 ºC	J. Am. Chem. Soc. 138, 14449-14457 (2016)
PCN-222	0.07 mM/h	6 mM	60 ºC	J. Am. Chem. Soc. 138, 14449-14457 (2016)
Microporous Metalloporphyrin-Containing Polymer	0.3 mM/h	20 mM	60 ºC	Angew. Chem. Int. Ed. 55, 6013-6017 (2016)
Cyclic Porphyrin Trimer	0.18 mM/h	9 mM	70 ºC	J. Am. Chem. Soc., 116, 3141-3142 (1994)
ZnPO-MOF	0.6 mM/h	6 mM	60 ºC	J. Am. Chem. Soc., 131, 4204-4205 (2009)
Monomeric Co-TPP	0.13 mM/h	6 mM	50 ºC	This work
Co-MMPG	0.70 mM/h	6 mM	50 ºC	This work
Co_2(DPX)	0.15 mM/h	6 mM	50 ºC	This work
Co_2(DPD)	0.17 mM/h	6 mM	50 ºC	This work
Co-MMPF	0.10 mM/h	6 mM	50 ºC	This work
MOF-525	0.46 mM/h	6 mM	50 ºC	This work

Comment 4: Is the work convincing, and if not, what further evidence would be required to strengthen the conclusions?

a. The results are partially convincing. The rate constants need to be extracted and compared with the relevant MOF system.

Response: The rate constants have been extracted and compared with the relevant MOF system. The other rate constants of porphyrin based catalytic system, such as microporous metalloporphyrin-containing polymer, porphyrinic dimer and trimer, have been also

summarized in Supplementary Table 1. As is shown in the table above (Response to comment 3), Co-MMPG shows superior activity to the relevant MOF system.

b. The NMR based proposed mechanism (electronic effect of the coordinated PC; Fig 4) is not convincing as the -OH group is not directly attached to the pyridine. Not sure what the authors wanted to convey in Fig4b. if it is various resonance structures drawn with the one-sided arrow it is not correct; if it represents various TS/intermediates then not sure what it means.

Response: We thank the reviewer for the comment. It has been reported that Lewis-acid activation is through the coordination of PC to the axial site of the metalloporphyrin to stabilize the positive charge on the oxygen (*J. Am. Chem. Soc. 2016, 138, 14449; J. Am. Chem. Soc. 2009, 131, 4204-4205*). In addition, it follows that the facilitation of the transition state due to complementary coordination may be aided by the "breathing" of the Gel bringing NAI and PC closer together and/or that some level of strain is permissible and necessary to form the transition state (also note that during anchoring N can only come as close to Co as the N−Co bond distance allows). Therefore, to gain further insight into the binding effect on substrate, a mixture of Co-TPP and 3-PC in DMSO-d_6 causes a shift of -CH_2 proton signal to a higher field, indicating the Co-TPP acts as an electron withdrawing group (Fig. 4a). A mixture of Co-TPP and 4-PC in DMSO-d_6 also causes a similar shift of -CH_2 proton signal to a higher field, however, the mixture of 2-PC with Co-TPP displays no shift mainly due to its steric effect from hydroxymethyl group. This difference further indicated the binding role of Lewis-acid activation through the coordination of 3-PC and 4-PC to the axial site of the Co-TCPP to stabilize the positive charge on the oxygen.

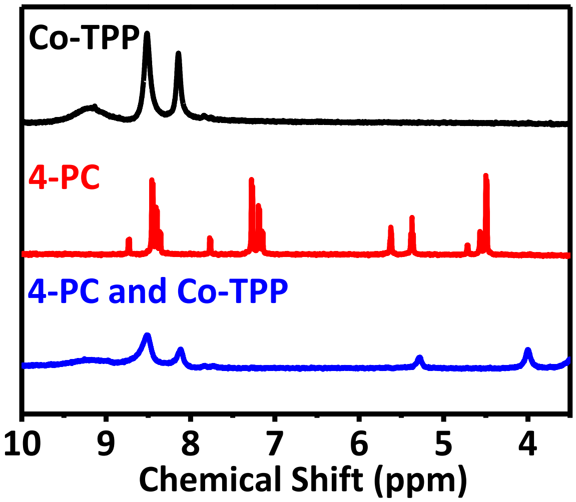

Supplementary Figure 28 | ^{1}H-NMR spectra of Co(II) meso-Tetraphenylporphyrin (Co-TPP) mixed with 4-pyridylcarbinol (4-PC) in DMSO-d_6 at room temperature

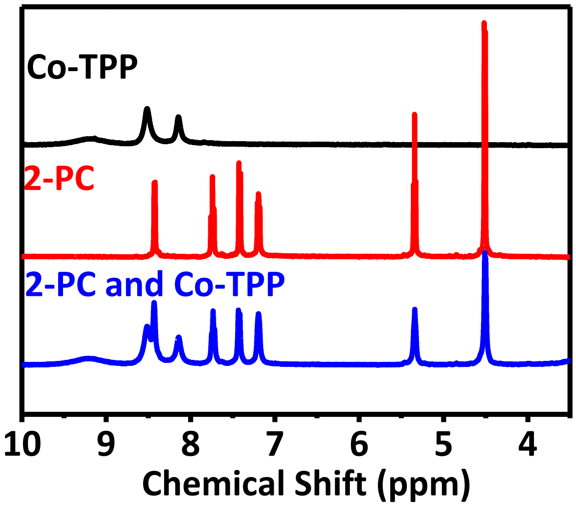

Supplementary Figure 29 | ^{1}H-NMR spectra of Co(II) meso-Tetraphenylporphyrin (Co-TPP) mixed with 4-pyridylcarbinol (2-PC) in DMSO-d_6 at room temperature

c. Scheme 1 does not clearly convey what is the backbone of the fiber if it involved an Al-O based polymer chain.

Response: We thank the reviewer for the comment. We have made clear the backbone of the fiber in Scheme 1.

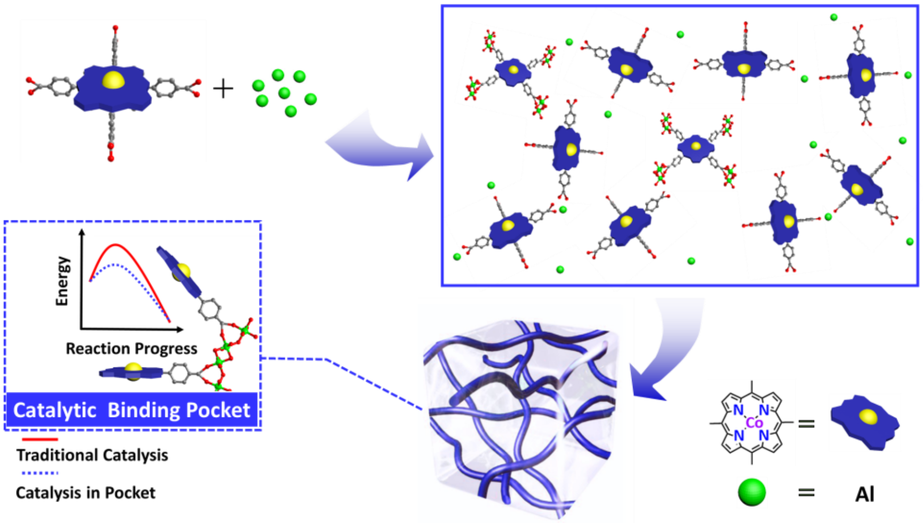

Scheme 1 | Schematic representation of the formation of Co-MMPG and its activation energy diagram with traditional catalysis and synergistic catalysis.

d. Not clear what is the H-bond system the authors mentioned several times to describe the structure.

Response: We thank the reviewer for the comment. To gain a better insight into the metal coordination gelation process, the UV-Vis absorption spectra of the Co-TCPP, Co-TCPP with $Al(NO_3)_3$ were measured in ethanol at temperatures ranging from 20-80 ^oC. We found slight difference in the absorption maxima for monomer forms of Co-TCPP versus Co-TCPP with $Al(NO_3)_3$. This suggests that there is a different electronic interaction between the H bond

system and metal coordination system of Co-porphyrin arrays. The change in UV-Vis absorption spectra for only Co-TCPP without $Al(NO_3)_3$ can be ascribed to the two-dimensional hydrogen-bonded network of Co-TCPP(*Adv. Mater. 2014, 26, 2072-2077, Angew. Chem. Int. Ed. 2015, 54, 500-505*). Therefore, the ligand-Al cluster based aggregate has a similar structure as the two-dimensional hydrogen-bonded network of Co-TCPP. This can help further analyze the coordination environment of Al and the Co-Co distance in Co-MMPG.

e. It is also not clear what would cause the change in 415 nm Soret band by altering 'aggregation'.
Response: We thank the reviewer for the comment. The formation of ligand-Al cluster based aggregate indicates that COOH groups coordinate with Al during the gel formation process. This causes the change that the intensity at 415 nm slowly goes up within increasing heating time up to 40 min.

f. ICP data suggest 1.0wt% Co content, so what is the point? Can these be changed in a controlled manner? How would the catalytic efficiency vary with that?
Response: We thank the reviewer for the comment. Inductively coupled plasma mass spectrometry (ICP-MS) together with elemental analysis indicated that the Co content is 1.0 wt%. The component of Co-MMPG is very close to that of Co-PMOF, but the Co content is a little bit lower. The Co content can be changed in a controlled manner *via* adjusting the ratio of Co-TCPP and TCPP during the gel formation process. However, a slight decrease of Co content leads to a sharp decrease of catalytic efficiency.

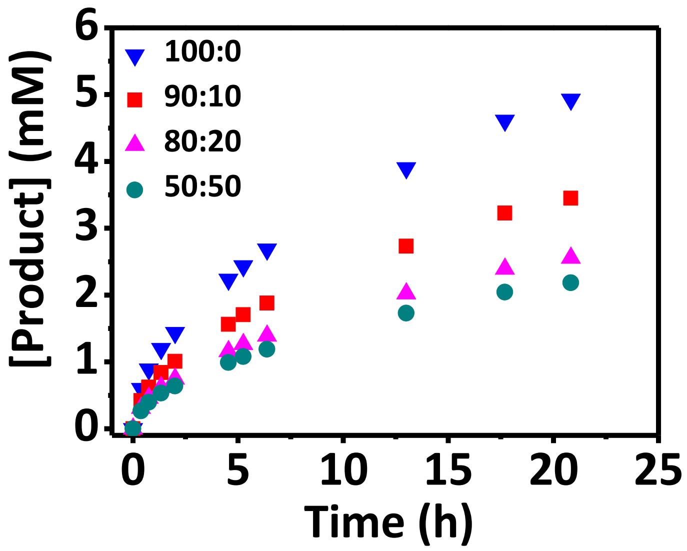

Supplementary Figure 15 | Catalytic property tests. Plot of product conversion versus time, showing the initial production of the various isomers catalyzed by Co-MMPG with various Co content (100 %, 90 %, 80 % and 50 %).

Comment 5: On a more subjective note, do you feel that the paper will influence thinking in the field? Not in the current form. The true potential of a new composition, such as MMPG, must establish a better and/or easier control of the structure-property to tie the performance (power to control the mechanism/reaction-pathway/ choice of substrate/stereogenic control of the product) to influence thinking in the field. On the contrary, the endo/exo DA reaction would be more interesting to explore/develop this work.

Response: We appreciate the reviewer for the constructive comment, especially the comment on our stereo-selective DA reaction catalyzed by gels. With the comments and questions raised by the reviewers fully addressed, we believe that our work will influence thinking in the fields of chemical science, materials science, catalysis science, energy science, etc. particularly when considering the following reasons.

Synergistic catalysis, as widely adopted by enzyme, is ubiquitous in biological systems. Extensive yet continuous efforts have been dedicated to mimic enzyme for synergistic catalysis, and significant progress has been accomplished in creating active center with binding/coordination environment similar to enzyme. Nonetheless, it remains a challenge, particularly in the solid state, to combine both binding pocket and conformational flexibility into one system to function like enzyme that features high specificity, high selectivity, and high efficiency. In this contribution we report the first aerogel capable of similar function by engineering multiple recognitions sites within the pocket, which is imperative for synergistic catalysis. The cooperativity between two Co(II) sites within the defined nanospace pocket facilitates the binding of different substrates with a favorable geometry thereby rendering Co-MMPG with excellent performance in the context of synergistic catalysis, especially for the kinetic control stereoselective reactions. Our work thus contributes a new enzyme-mimic design strategy to develop highly efficient heterogeneous catalyst with high chemo/stereo selectivity.

In addition, Metal-organic gels (MOGs), a new type of functional porous material-aerogels possessing versatile porosity, low density and high internal surface area properties, can easily be used to make monolithic solids of desired shapes and is proven to work for applications such as heterogeneous catalysis and adsorption/separation technology, and energy application. Although MOGs offer new opportunities for new-generation materials with improved or new synergetic properties not found in their individual components, it is still a challenge to understand the coordination environment of metals, local structure of gels and relationship between structure and performance. Our design strategy herein provides a new approach to construct metal-organic gel materials and other soft porous materials with synergistic/cooperative effect for a broad range of applications, such as catalysis, gas storage/separation, etc.

Moreover, synergistic catalysis occurring in an enzyme pocket shows enhanced performance through supramolecular recognition and conformational adaptations. The metalloporphyrin-based metal-organic gel featuring catalytic binding pocket to mimic enzymes has been achieved for the first time by combining cobalt porphyrin monomers with a twisting Al-O-Al cluster. As a result of the synergy between the Lewis acid centers as binding sites and defined nanospace pockets, Co-MMPG demonstrates excellent performance in the context of synergistic catalysis, especially for the kinetic control stereoselective reactions. Our work thereby not only provides a new enzyme-mimic design approach to prepare highly active MOG-based catalysts but also lays a solid foundation for the development of metal-organic gel as a new type of highly efficient heterogeneous catalyst that can merge synergistic catalysis and porous materials to carry out chemo/stereo-selective chemistry.

Furthermore, the binding pocket conformation in Co-MMPG was elucidated through the combined studies of solid-state NMR and X-ray absorption near-edge structure (XANES). The cooperativity between two Co(II) sites within the defined nanospace pocket renders Co-MMPG with interesting synergistic catalysis as illustrated in the context of acyl –transfer and Diels—Alder reactions. Specifically, Co-MMPG demonstrated the most efficient catalytic activity for acryl-transfer of the compounds studied in terms of conversion particularly with a ~10-fold enhancement in reaction rate compared to the monomer, which can be presumably attributed to the binding event in its bimolecular-like pocket. Moreover, the product selectivity, such as reversing the stereochemical outcome of a Diels-Alder reaction, favored the *endo* over the *exo* adduct product has also been achieved. Co-MMPG therefore suggests a new enzyme design strategy to improve chemo/stereo-selectivity in catalysis.

Again we thank all reviewers for the constructive comments and suggestions, which have made our manuscript much improved.

Sincerely,

Shengqian Ma, PhD
Professor of Chemistry

REVIEWERS' COMMENTS:

Reviewer #1 (Remarks to the Author):

I appreciate the authors' effort in revising the manuscript and making point-by-point responses to the two reviewers. I have no more suggestions and the current version is recommended for publication in Nature Communication.

Reviewer #2 (Remarks to the Author):

The modified version appears significantly improved. The authors have addressed the majority of the original concerns. However, there are still some issues that need to addressed/discussed in a minor revision process.

1) (a) While establishing the novelty, the authors point out flexibility. Without defining it- it remains vague in the manuscript. The authors also argued the original reviewer's concern with "it is essential to be flexible but not too flexible, as well as rigid but not too rigid". It is recommended to make this point on the 'optimal flexibility' discussed in the manuscript with a comment stating how the current MOG system imparts that better than MOF.

(b) The authors estimated a range of Co-Co distance 6.8-16.7 A; which means not all will be effective? And it may be the case that, at least, partially the activity is due to the wide range of available Co-Co distance and some better fit the required one (in contrast to that limited availability in MOF) where 'flexibility is not the sole reason, as claimed.

2) (a) The authors compared their result with the literature values. Supplementary table 1 reports 'concentration' –this is for the substrate? Please clarify.

(b) The LA catalyzed acyl transfer reaction depends on the synergy (binding both the reagent/substrates in favorable geometry) and the concentration of them achieved within the porous catalysts determined roughly by the concentration of the active site. The ICP suggest 1% Co: from this and the ratio of the Al:Co (and other values) can the authors deduce the concentration of their active site. Only then the Sup Table 1 will be complete to compare with the MOF catalysts.

3) It is recommended to remove Fig 4 and relevant text. The 1H NMR shift of the 3-PC and 4-PC upon coordination to the Co-TCPP is most likely due to the aromatic ring current effect. Such effect is known to cause an up-field shift for coordinated species (sometimes 4 to -1 ppm; see, as one example – JACS, 1997, 119, 12362). It is clear from the Sup Fig 28 and Fig 4a that some, if not all, of the aromatic H peaks for the coordinated Pyridine, has moved up-field. However, 2-pc, due to steric hindrance will have low binding constant and thus all the peaks remain unchanged (Sup. Fig. 29). Figure 4b shows two negative charges: where from? What reduced the Pyridine – one of the forms that put the negative charge on the –methylene (-CH2-) moiety –how the valency is accounted? Perhaps the authors wanted to show that N(py) ligation to the LA Co(III) induce some positive charge and it surely will be least at the 3-position which will destabilize the least the delta+ charge on the 'O' at the TS/intermediate.

4) The UV-vis analysis still remains confusing. The H-bonding (unclear what constitute that – who is H-bond donor and who is acceptor), aggregation, and COOH-coordination to the Al center. It is unlikely that just Al-binding the sidearm -Ph-COOH will cause any blue shift. It is possible that H-type

aggregation/stacking which indicates the desired structure formation. Please describe specifically what is the fundamental reason, not a hand waving assignment.

Dr. Shengqian Ma
Professor

Department of Chemistry
University of South Florida
4202 E. Fowler Avenue, CHM 205A
Tampa, FL 33620

Phone: 1-813-974-5217
Fax: 1-813-974-3203
E-mail: sqma@usf.edu
Website: http://sqma.myweb.usf.edu/

Date: March 31, 2019

We greatly appreciate the constructive comments and suggestions from both reviewers, and we have revised the manuscript accordingly as detailed in the responses below. The corresponding changes have been highlighted in yellow in the main text and Supplementary Information.

Reviewer #1:

I appreciate the authors' effort in revising the manuscript and making point-by-point responses to the two reviewers. I have no more suggestions and the current version is recommended for publication in Nature Communication.

Response: We thank the reviewer's support of our work for publication in Nature Communications.

Reviewer #2:

The modified version appears significantly improved. The authors have addressed the majority of the original concerns. However, there are still some issues that need to addressed/discussed in a minor revision process.

Response: We thank the reviewer for taking the time to review our manuscript again. Per the reviewer's suggestion, we further revise the paper to address the following comments.

Comment 1: (a) While establishing the novelty, the authors point out flexibility. Without defining it- it remains vague in the manuscript. The authors also argued the original reviewer's concern with "it is essential to be flexible but not too flexible, as well as rigid but not too rigid". It is recommended to make this point on the 'optimal flexibility' discussed in the manuscript with a comment stating how the current MOG system imparts that better than MOF.

Response: We appreciate the reviewer for the constructive comment. As the reviewer suggested, we demonstrate the point on the 'optimal flexibility' discussed in the manuscript with a comment stating how the current MOG system imparts that better than MOF.

(b) The authors estimated a range of Co-Co distance 6.8-16.7 A; which means not all will be effective? And it may be the case that, at least, partially the activity is due to the wide range of available Co-Co distance and some better fit the required one (in contrast to that limited availability in MOF) where 'flexibility is not the sole reason, as claimed.

Response: Yes. As the reviewer mentioned, not all Co-Co distances are effective for catalysis. It has been previously reported that the highest initial rate for 3-PC with MOF-525 is about double that of the reported NU-902 (*J. Am. Chem. Soc. 2016, 138, 14449-14457*). Therefore, it can be deduced that Co-Co distance of no less than 10 Å together with favorable orientation and proximal positioning of NAI and PC by metal sites favors the rate enhancement. Obviously, the optimal flexibility of MOGs brings a wide range of Co-Co distance with favorable orientation and proximal position of NAI and PC by metal sites.

Comment 2: (a) The authors compared their result with the literature values. Supplementary table 1 reports 'concentration' –this is for the substrate? Please clarify.
Response: We are thankful to the reviewer for the comments. As the reviewer mentioned, the "concentration" in Supplementary table 1 means the concentration of N-acetylimidazole.

(b) The LA catalyzed acyl transfer reaction depends on the synergy (binding both the reagent/substrates in favorable geometry) and the concentration of them achieved within the porous catalysts determined roughly by the concentration of the active site. The ICP suggest 1% Co: from this and the ratio of the Al:Co (and other values) can the authors deduce the concentration of their active site. Only then the Sup Table 1 will be complete to compare with the MOF catalysts.
Response: We are thankful to the reviewer for the comments. The concentration of metal sites for catalysts summarized in Sup table 1 has been provided.

Comment 3: It is recommended to remove Fig 4 and relevant text. The 1H NMR shift of the 3-PC and 4-PC upon coordination to the Co-TCPP is most likely due to the aromatic ring current effect. Such effect is known to cause an up-field shift for coordinated species (sometimes 4 to -1 ppm; see, as one example –JACS, 1997, 119, 12362). It is clear from the Sup Fig 28 and Fig 4a that some, if not all, of the aromatic H peaks for the coordinated Pyridine, has moved up-field. However, 2-pc, due to steric hindrance will have low binding constant and thus all the peaks remain unchanged (Sup. Fig. 29). Figure 4b shows two negative charges: where from? What reduced the Pyridine – one of the forms that put the negative charge on the –methylene (-CH2-) moiety –how the valency is accounted? Perhaps the authors wanted to show that N(py) ligation to the LA Co(III) induce some positive charge and it surely will be least at the 3-position which will destabilize the least the delta+ charge on the 'O' at the TS/intermediate.
Response: We are thankful to the reviewer for the criticism. Fig 4 and relevant text have been removed.

Comment 4: 4) The UV-vis analysis still remains confusing. The H-bonding (unclear what constitute that – who is H-bond donor and who is acceptor), aggregation, and COOH-coordination to the Al center. It is unlikely that just Al-binding the sidearm -Ph-COOH will cause any blue shift. It is possible that H-type aggregation/stacking which indicates the desired structure formation. Please describe specifically what is the fundamental reason, not a hand waving assignment.
Response: We thank the reviewer for the comment. Hydrogen bonds are known to form between carboxyl groups (Spectrometric Identification of Organic Compounds. 8th ed. John Wiley and Sons; Hoboken, NJ, USA: 2005.). In this case, the change in UV-Vis absorption spectra for only Co-TCPP without $Al(NO_3)_3$ can be ascribed to the two-dimensional hydrogen-bonded network of Co-TCPP(*Adv. Mater. 2014, 26, 2072-2077, Angew. Chem. Int. Ed. 2015, 54, 500-505*).

Again we thank the reviewers for the constructive comments and suggestions, which have made our manuscript further improved.

Sincerely,

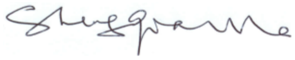

Shengqian Ma, PhD
Professor of Chemistry
